# Electrically Tunable Piezotronic Transistor by Coupling Interface Polar Symmetry and Strain Gradient

**DOI:** 10.1002/advs.76341

**Published:** 2026-07-03

**Authors:** Gongwei Hu, Xiaoming Dong, Yihan Zhang, Yong Chao, Menglu Li, Min Liu, Liqing Pan, Lijie Li, Wei Huang, Fobao Huang

**Affiliations:** ^1^ Hubei Engineering Research Center of Weak Magnetic‐Field Detection College of Mathematics and Physics China Three Gorges University Yichang China; ^2^ State Key Laboratory of Flexible Electronics (LoFE) & School of Integrated Circuits (School of Microelectronics) Northwestern Polytechnical University Xi'an China; ^3^ Shenzhen Research Insititute of Northwestern Polytechnical University Shenzhen China; ^4^ Yangtze River Delta Research Institute of Northwestern Polytechnical University Taicang China; ^5^ Faculty of Science and Engineering Swansea University Swansea UK

**Keywords:** bulk piezoelectric charges, interface polar symmetry, multifunction sensing, strain gradient, tunable piezotronic effect

## Abstract

Piezotronics, which enables mechanical stimuli to actively shape adaptive and seamless interactions between electronic systems and ambient environments, is becoming increasingly valuable in the Internet of Things, human‐machine interfaces, and wearable electronics. Interface‐dominated polarization underpins efficient electromechanical transduction in piezotronic sensors; however, it enforces limited performance tunability and hampers multifunctional applications. Here, we use a macroscale tip‐induced strain gradient to trigger a bulk‐dominated polarization in GaN for realizing a tunable piezotronic effect. Such a mechanism uses interface polar symmetry and shielding of bulk piezo‐charges to drive an electrical switching between high‐ and low‐strain sensitivity states. The tunability is preserved across different indenter sizes, while spherical tips with larger radii further enhance the modulation and tuning range due to strain‐gradient size effects. Our piezotronic device has a wide sensitivity tunability window of 24 403, a large pressure sensitivity of 223.2 meV·MPa^−1^, and ultrahigh strain sensitivity of 1.43 × 10^8^. Moreover, the piezotronic device shows exceptional mechanical durability up to 10 000 loading cycles and preserves electrical tunability even under dynamic operation. This study enriches piezotronic physics by uncovering the cooperative roles of bulk polarization and interface symmetry in transport modulation, and establishes a viable strategy for continuous and wide‐range performance tunability in a single mechanical sensor.

## Graphical abstract

A macroscale tip‐induced strain gradient generates bulk piezo‐charges in GaN, enabling bias‐controlled shielding and selective Schottky barrier modulation. This strain‐gradient piezotronic transistor exhibits electrically switchable high‐ and low‐sensitivity states, providing ultrahigh strain sensitivity and wide tunability for adaptive mechanical sensing beyond conventional interface‐dominated piezotronics.

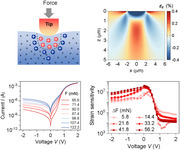

## 1 Introduction

Electronic systems that can directly couple mechanical stimuli with electrical functions form a fundamental building block for many emerging technologies, including the Internet of Things, human–machine interfaces, wearable electronics and intelligent robots [1, 2, 3, 4, 5, 6, 7, 8, 9]. In most conventional sensor architectures, mechanical deformation acts only as a passive perturbation that weakly tunes pre‐defined electrical characteristics. Such limitation has motivated growing interest in designing advanced electromechanical paradigms, in which mechanical signal is elevated to an active control variable in electronic devices [1, 4, 7, 9]. In this context, the piezotronic effect has emerged as a brand‐new frontier for mechanically controlled electronics that uses strain‐induced piezoelectric polarization charges (piezo‐charges) and the associated piezoelectric potential to directly modulate barrier and thus actively control carrier transport across interface of metal‐semiconductor, semiconductor‐semiconductor and insulator‐semiconductor junctions [2, 10]. Due to such inherent coupling of piezoelectricity and transport properties, sensors based on the piezotronic effect usually exhibit efficient signal transduction between mechanical stimulus and electrical response, causing asymmetric carrier transport, exceptional sensitivity, and exponential current amplification [11, 12, 13, 14]. These features make piezotronic effect particularly promising for the applications in tactile imaging [1], and force‐mapped light‐emitting diodes [15], ultrasensitive pressure and strain sensors [7, 9, 11, 12, 13, 14, 16, 17, 18], and emerging quantum sensing technologies [19, 20].

**FIGURE 1:**
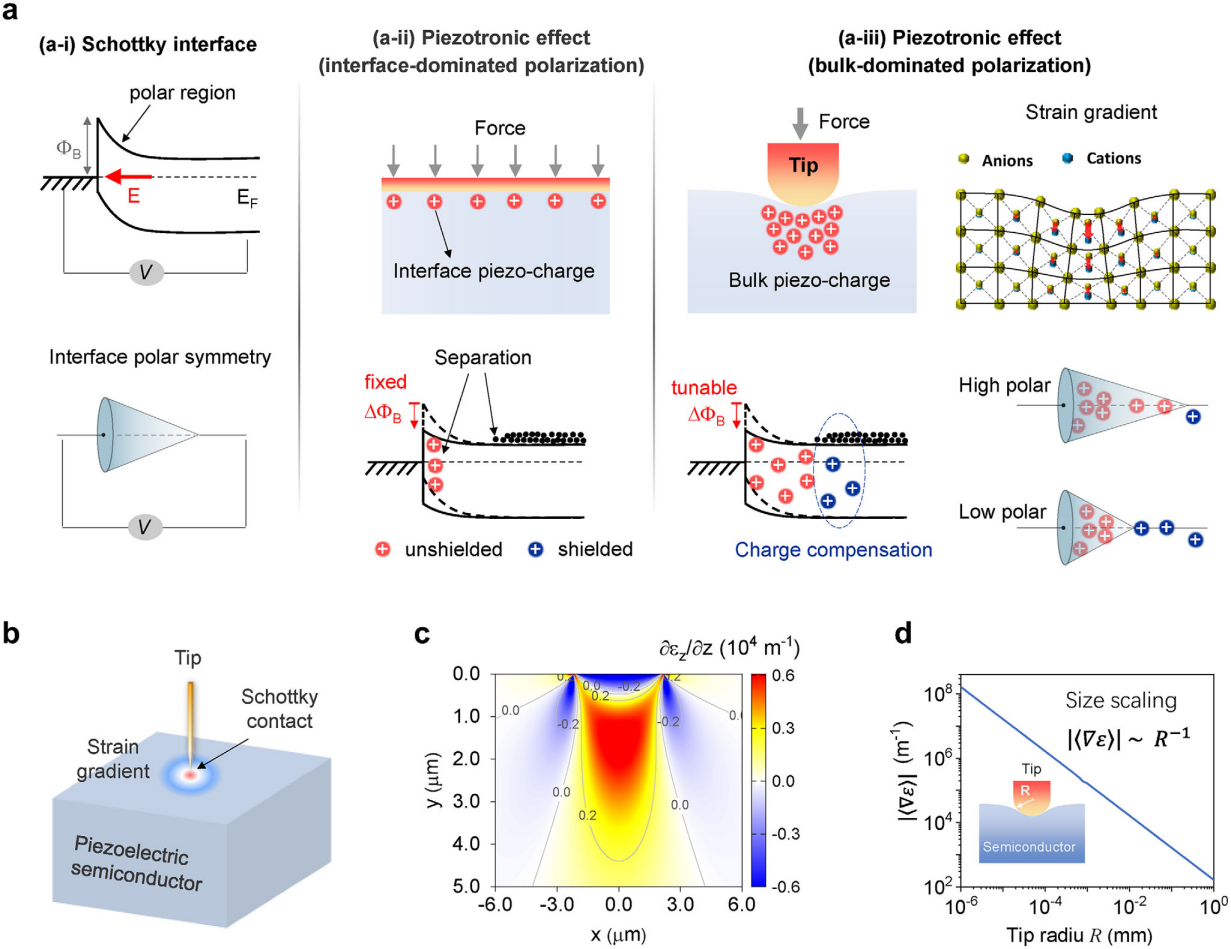
The working mechanism of piezotronic effect under interface polar symmetry and strain gradient. (a) Interface polar symmetry, and piezotronic effect with interface‐ and bulk‐dominated polarization mechanisms. At a Schottky interface, the interface polar symmetry is induced by a carrier‐depleted electric field with a cone‐like profile that is tunable by bias voltage (a‐i). When a uniform strain is imposed on a piezoelectric semiconductor, the piezo‐charges are generated at interface to reduce Schottky barrier height by ΔΦ_B_. Interface piezo‐charges are separated from the free electrons without direct charge compensation, causing fixed ΔΦ_B_ (a‐ii). For the case of tip‐induced strain gradient, bulk piezo‐charges are produced inside materials and could be partially shielded by free electron at the end of polar region due to the charge compensation, which is a bulk‐dominated polarization mechanism. The number of unshielded piezo‐charges are determined by the interface polar region with a bias‐controllable width, causing a tunable ΔΦ_B_ (a‐iii). (b) Schematic diagram of a strain‐gradient piezotronic transistor. (c) The calculated distribution of strain‐gradient ∂ε_*z*_/∂*z* generated by a spherical indenter with a radius of 50 µm under a load of 100 mN. (d) The absolute value of the averaged strain gradient as a function of the tip radius (inset). By fitting the curve, the strain gradient meets the size scaling law $\left|\left\langle \nabla \varepsilon \right\rangle \right|∼R^{-1}$.

In electronic devices, the interface barrier plays a crucial role in determining the transport, functionalities, and even application scenarios [2, 21]. Until now, the piezotronic effect has been predominantly realized in Schottky [22, 23, 24, 25] and tunneling junctions [7, 9, 11, 12, 13, 14], where signal transduction is governed by interface‐dominated polarization. When external strain is exerted on a piezoelectric semiconductor, piezoelectric polarization is induced to generate interface piezo‐charges for tuning of the local electric field and barrier height [10, 26]. Meanwhile, an inherent electric field (for example, the depletion field in a Schottky contact, Figure 1a‐i) breaks interface symmetry to establish a polar region that spatially separates interface piezo‐charges from mobile carriers (Figure 1a‐ii), suppressing electrostatic screening and producing strong barrier modulation, and thus high sensitivity [26]. Despite the strong electromechanical coupling, the lack of direct charge compensation makes the shielding difficult to control by electrical means. Therefore, interface barrier typically follows a single, predetermined modulation mode with limited tunability of piezotronic effect, falling short of delivering the continuous performance adjustability that is increasingly desired for adaptive, multifunctional, and reconfigurable mechanical sensing [1, 2, 27, 28]. To overcome this limitation, considerable efforts have been devoted to designing materials and device architectures, including electric pulse‐induced rearrangement of oxygen vacancies to modify barrier height in ZnO nanowire [9], switching the transport regimes between thermionic emission and tunneling [16, 17, 18], engineering surface states at a tunneling junctions [11, 13], and manipulating the piezoelectricity at polar interface [29]. Although these methods have yielded incremental tunability, they often introduce trade‐offs such as restricted tuning range, unstable responses, or reduced sensitivity [9, 16, 17]. Therefore, finding a strategy that enables continuous and stable tuning of the piezotronic effect is of significance for achieving more functional flexibility in a single mechanical sensor and expanding the applications of electronics.

Beyond interfacial piezoelectric polarization, mechanical deformation can also regulate the interface barrier through bulk polarization generated by strain gradients [4, 8, 30, 31, 32]. When strain is spatially nonuniform, local inversion symmetry is broken, and flexoelectric polarization emerges even in centrosymmetric materials [33]. Such a strain gradient provides a volumetric source of polarization‐bound charges distributed inside a material [34]. Owing to its universal presence and geometric amplification at small scales, flexoelectricity has inspired rapidly expanding interest in the flexo‐photovoltaic effect [35] and triboelectricity [31], photodetectors [36], current rectification [32], and mechanical sensing [4, 8, 30]. Despite these advances, the practical implementation of flexoelectric devices for mechanical sensing remains challenged on several fronts. On the one hand, achieving sufficiently large strain gradients typically requires nanoscale contact geometries, such as atomic force microscope probes, which inherently introduce pronounced size effects in both strain distribution and device response [34]. Such nanoscale tips are prone to wear and mechanical damage during prolonged operation, resulting in strong dependence on tip sharpness, unstable performance, and limited robustness for practical packaging. On the other hand, most centrosymmetric semiconductors such as Si and SrTiO3 possess low flexoelectric coefficients that restrict electromechanical coupling strength and lead to limited sensing sensitivity [4, 8, 30, 32].

In this article, an ultrahigh‐sensitivity and electrically tunable piezotronic sensor is achieved by using a macroscale tip‐induced strain gradient to induce bulk piezo‐charges in a piezoelectric GaN film. The synergy between the interface polar symmetry and the shielding of bulk piezo‐charges enables selective modulation of Schottky barrier height by bias voltage, yielding the electrical switching of high and low strain sensitivity. By optimizing structures, the piezotronic device exhibits a wide tunable sensitivity window of 24 403, a large pressure sensitivity of 223.2 meV·MPa^−1^, and an ultrahigh strain sensitivity of 1.43 × 10^8^, five orders of magnitude higher than those of flexoelectric devices. The electrical tunability of our piezotronic sensor is universal across different indenter geometries and sizes, while spherical tips and larger radii further amplify the modulation and tuning range due to the size effect of the strain gradient. In addition, the piezotronic sensor exhibits excellent durability even over 10 000 repeated cycles and preserves electrical tunability even under dynamic load. This study not only enriches piezotronic physics by revealing how bulk‐dominated piezoelectric polarization and interface symmetry breaking cooperate to modulate transport, but also offers an effective route to achieve continuous and highly tunable sensing performance in a single mechanical sensor, which opens opportunities for intelligent and integrated sensing applications.

## 2 Results

### 2.1 Interface Polar Symmetry and Strain Gradient

In terms of symmetry breaking, electric fields and strain gradients play analogous roles [37, 38]. Taking the built‐in electric field at a Schottky interface as an example, this field is a vector pointing toward the interface and exhibits a cone‐like polar symmetry [39, 40]. By tuning the electric field using a bias voltage, the polar symmetry becomes tunable (Figure 1a‐i). On the other hand, under mechanical loading, a tip generates a strain gradient that distorts the atomic arrangement and breaks inversion symmetry [33] (Figure 1a‐iii). If the material is piezoelectric, such a strain gradient could induce an inhomogeneous piezoelectric polarization with bulk piezo‐charges, which are distributed over a finite depth beneath the contact. At the end of the polar region of the Schottky interface, these bulk piezo‐charges can be electrostatically shielded by the free carriers due to their direct charge compensation, and only the unshielded piezo‐charges modulate the interface barrier. Hence, the barrier modulation ΔΦ depends on the width of the polar region or polar symmetry strength, which are bias‐tunable. Concretely, for the strong polar case, massive bulk piezo‐charges populate the polar region with an electric field that repels free electrons, causing weak electrostatic shielding and thus larger ΔΦ. This modulation is suppressed when a large number of piezo‐charges are repelled outside the polar region by reducing the interface electric field. Such unique piezotronic effect stems from bulk‐dominated polarization charges due to strain gradient, and differs from conventional piezotronic modulation where interface piezo‐charges are separated from free electrons without a direct charge compensation (see Figure 1a‐ii).

### 2.2 Tip‐Induced Strain Gradient in a Piezoelectric Semiconductor

A hard‐conductive tungsten (W) tip, subjected to a load force, is used to generate a strain gradient on n‐GaN piezoelectric semiconductor (Figure 1b). According to Hertzian contact theory [41], this tip generates high non‐uniform strain and strain gradient (see Note S1 and Figure S1). As a demonstration, Figure 1c shows the distribution of strain gradient ∂ε_z_/∂z for a spherical tip radius of *R* = 50 µm under a load F = 100 mN. The ∂ε_z_/∂z is negative near the contact but positive away from the surface, showing a magnitude of 10^4^ m^−1^. Owing to the large tip radius, this strain gradient is not so high compared to the case of an atomic force microscope (AFM) tip (∂ε_z_/∂z ∼ 10^7^ m^−1^ at R < 100 nm) [39].

We calculate an averaged strain gradient |⟨∇ε⟩| (see Note S2) as a function of tip radius *R* (Figure 1d and inset). As we can see, the |⟨∇ε⟩| is high at the nanoscale but decreases rapidly with increasing *R*, scaling as |⟨∇ε⟩| ∼ *R* ^−1^. Such a scaling property arises from geometric effects [34]. Taking an example, for two inclusions of identical aspect ratio but different sizes, the strain field remains constant, while the strain gradient scales inversely with the characteristic length (see Figure S2). The scaling law could cause the strain gradient to be sensitive to the fluctuation of the tip radius at nanoscale but insensitive at our macroscale tip (see Note S2 and Figure S2).

### 2.3 Tip‐Induced Tunable Piezotronic Effect

**FIGURE 2:**
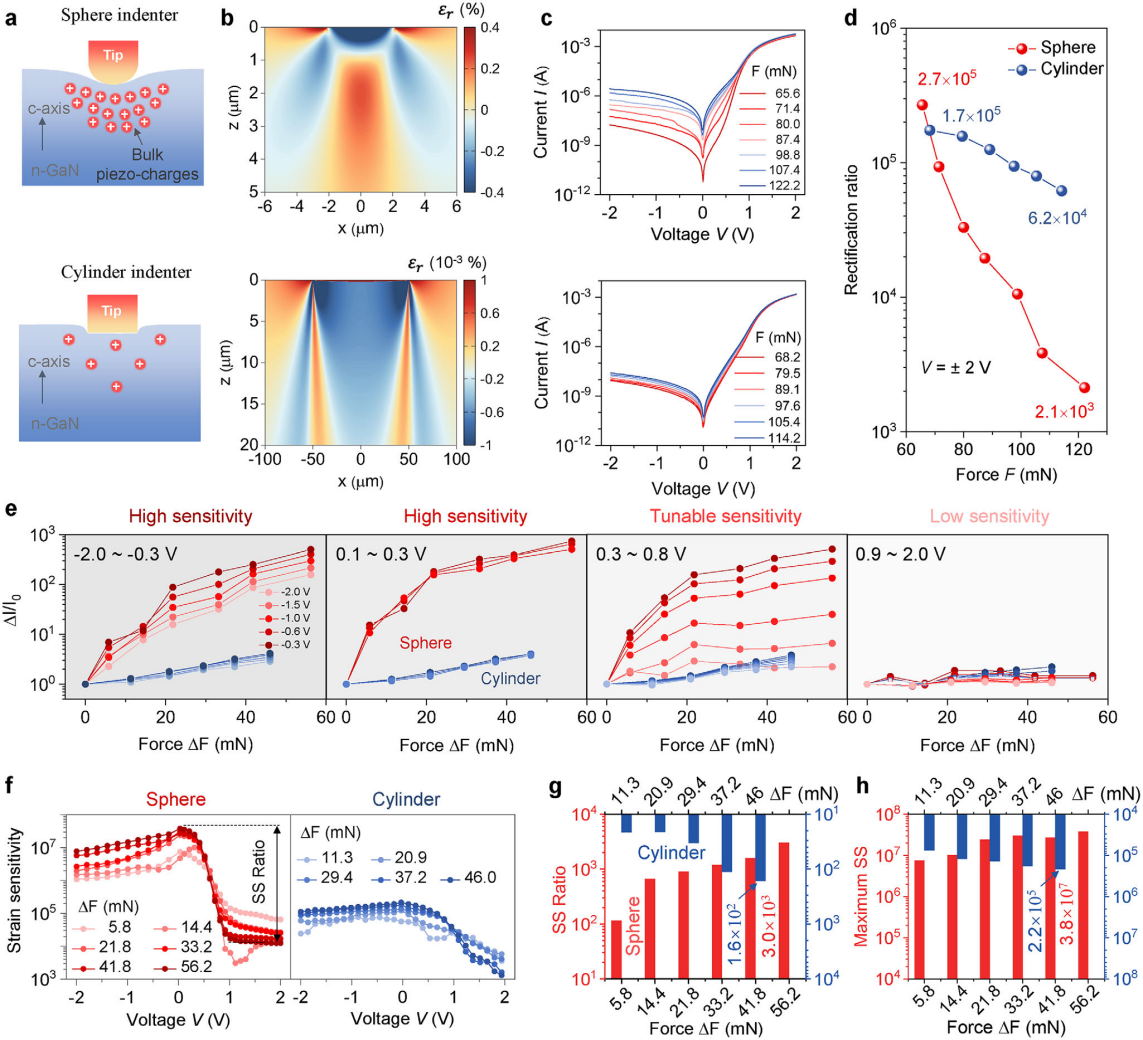
The electrically tunable piezotronic response in GaN‐based SGPT under spherical and cylindrical indenters. (a) The schematic diagram of bulk piezo‐charges beneath the n‐GaN surface. (b) The calculated distributions of strain ε_*r*_ for tip indenter (radius R = 50 µm) under a load F = 100 mN. (c) Logarithmic current‐voltage (I‐V) characteristics at different loads. In (a–c), the cases of spherical (upper) and cylindrical (lower) indenters are shown. (d) Rectification ratio (at *V* = ±2 V) as a function of load for both indenters. (e) ΔI/I_0_ versus load for spherical (red) and cylindrical (blue) indenters at different bias ranges, including high‐sensitivity (−2 < *V* < −0.3 V, leftmost), high‐sensitivity (0< *V* < 0.3 V, second from left), tunable‐sensitivity (0.3 < *V* <0.8 V, second from right), and low‐sensitivity (0.9 < *V* < 2.0 V, rightmost) regimes. (f) Bias‐dependent strain sensitivity (SS) for spherical (left) and cylindrical (right) indenters. (g) The SS ratio, and (h) maximum SS.

We study a rigid W tip (R = 50 µm) exerting a normal load on an n‐GaN thin film (see Methods). The W tip imposes a strain gradient and forms a Schottky contact with n‐GaN. Because the strain‑gradient magnitude depends on tip geometry [41], we first compare the cases of spherical (upper) and cylindrical (lower) tips in Figure 2a. Under a load, the spherical indenter produces a larger strain gradient than the cylindrical case, and thus more bulk piezo‐charges. This is due to the cylindrical indenter with a wide contact surface that causes lower strain (Figure 2b).

We measure the current–voltage (*I–V*) characteristics of strain‐gradient piezotronic transistor (SGPT) based on spherical (upper) and cylindrical (lower) tips in Figure 2c. A tip‐loading stage is used here to apply and monitor the load force (see Method and Figure S3). Both SGPTs are single‐side Schottky consisting of a W tip (radius *R* = 50 µm) and a coated Al Ohmic electrode (Figure S4). The results show clear load‐dependent current under reverse bias, whereas the forward‑bias response diminishes as the voltage increases. This bias asymmetry is particularly evident for the spherical tip, where the rectified ratio at *V* = ±2 V is reduced by 128.6, far higher than the cylindrical case (see Figure 2d).

To compare piezotronic modulation, we plot the relative current change ΔI/I_0_ = [I(F) − I_0_]/I_0_ versus the load increment ΔF = F − F_0_ at different biases (Figure 2e). Here, F_0_ is 65.6 mN for the spherical tip and 68.2 mN for the cylindrical case. Three sensitivity regimes are observed, including high sensitivity (−2.0 to −0.3 V and 0.1 to 0.3 V), tunable sensitivity (0.3–0.8 V), and low sensitivity (0.9–2.0 V). In the highly sensitive regime, ΔI/I_0_ is large and increases with reverse bias but remains bias‐independent in the forward case (0.1–0.3 V). For higher forward bias, ΔI/I_0_ drops markedly with *V*, and then saturates to a low value for *V* > 0.9 V. These features appear for both spherical and cylindrical indenters. However, the spherical indenter exhibits more evident bias‐dependent response, confirming stronger piezotronic modulation.

Figure 2f plots bias‐dependent strain sensitivity (SS) for SGPTs under different loads. As expected, the SS remains high and stable at reverse bias, then drops sharply at small forward bias and settles at a low plateau. Within bias of −2–2 V, the ratio between the maximum and minimum SS, evaluating the tunability of sensitivity, increases with load for both tips (Figure 2g). The maximum SS ratio reaches 3.0 × 10^3^ for the spherical indenter (at Δ*F* = 56.2 mN) and 1.6 × 10^2^ for the cylindrical case (at Δ*F* = 46.0 mN), which indicates excellent tunability in sensing performance. Moreover, the spherical indenter has a higher strain sensitivity with peak SS of 3.8 × 10^7^, two orders of magnitude higher than that of the cylindrical case (Figure 2h).

To confirm the reversibility of the bias‐dependent sensitivity switching, we performed forward‐and‐backward bias‐sweep measurements under different loading forces (see Figure S5). The *I–V* curves show good overlap during the forward and backward sweeps, with no obvious electrical hysteresis. The extracted I–load curves at representative voltages from −2 to 1 V also exhibit good consistency between the two sweeping directions. These results confirm that the switching between high‐ and low‐sensitivity states is reversible and reproducible.

### 2.4 The Effect of Surface Polar Symmetry

**FIGURE 3:**
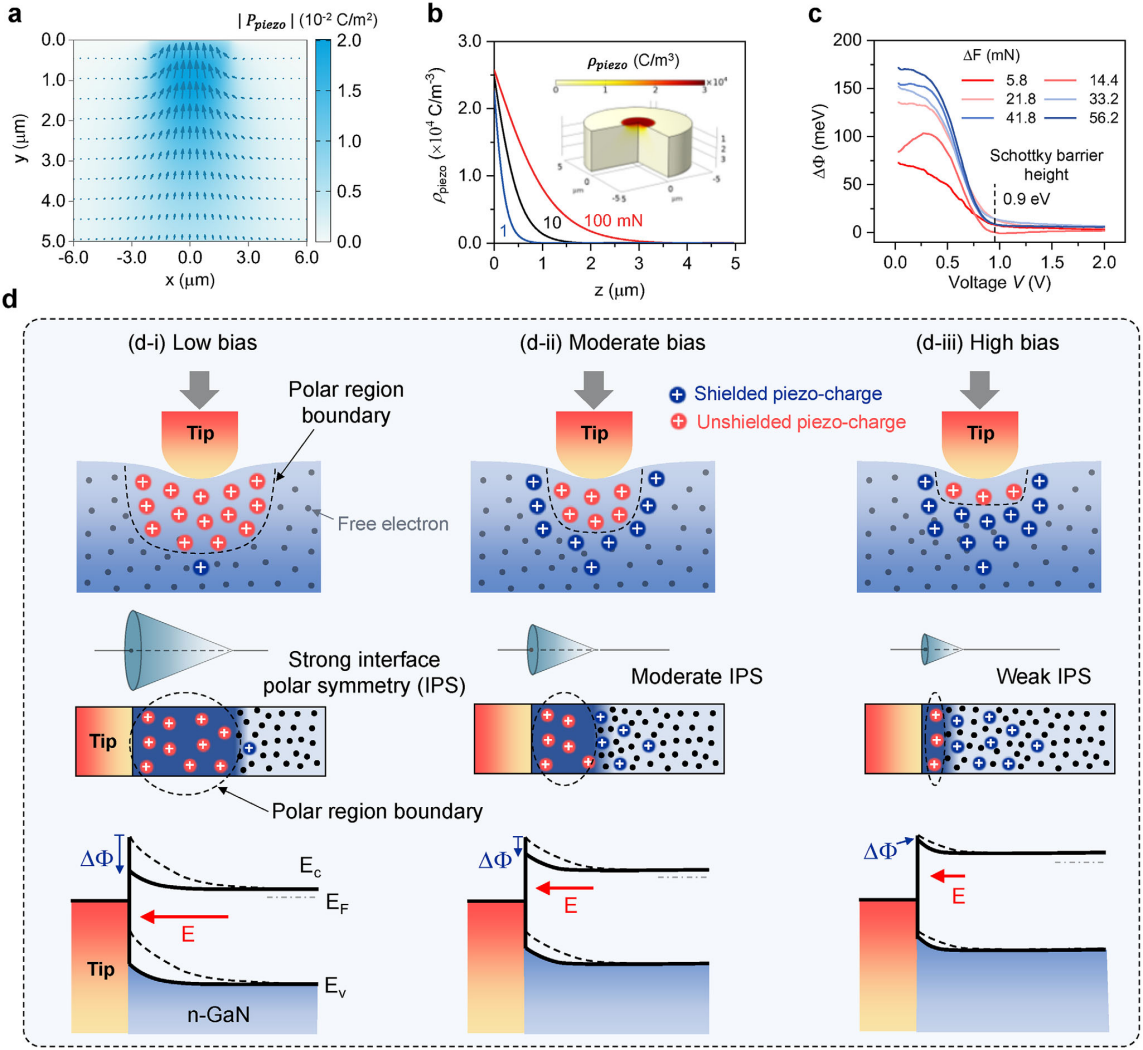
The combined effect of surface polar symmetry and electron shielding. (a) Distribution and direction (arrows) of the calculated piezoelectric polarization. (b) The bulk piezo‐charges along the center axis, and the 3D profile (inset). In (a, b), the indenter radius is R = 50 µm and the applied force is F = 100 mN. (c) Bias‐dependent barrier height change ΔΦ under different loads. (d) The working mechanism of bias‐dependent piezotronic response. At low bias (a‐i), the large electric field E at the Schottky interface causes a strong interface polar symmetry (IPS) with a wide polar region that fully encloses the bulk piezo‐charges, leading to a pronounced modulation of barrier height by load. As the bias increases with a moderate IPS (a‐ii), the polar region narrows, and part of the piezo‐charges lies outside this region and is shielded by free electrons, which weakens the barrier modulation. Such modulation is further suppressed at a high bias (a‐iii), where the polar region almost vanishes with weak IPS and most of the piezo‐charges are shielded.

We calculate the piezoelectric polarization and the resulting charge distribution (see Note S1). Figure 3a shows the absolute value of total piezoelectric polarization | ***P**_piezo_* | under a load of F = 100 mN. The polarization is confined to a narrow region with the direction pointing toward the interface, which leads to positive bulk piezo‐charges ρ_*piezo*_ =   − ∇ · ***P**_piezo_* (the inset of Figure 3b). The ρ_*piezo*_ is maximum at the contact interface, and rapidly reduced along the center axis (Figure 3b). Increasing the applied load does not substantially change the peak ρ_*piezo*_, but extends the charge region.

For a Schottky device, the barrier height is the key parameter governing the transport. According to the thermionic emission theory [42], the change in Schottky barrier height (SHB) can be evaluated as Δϕ  =  *kT* ln (I/I_0_), where, *I*_0_ denotes the current under a reference load *F*_0_, *I* is the current under an applied load *F*, ΔΦ = Φ(*F*)—Φ(*F*_0_) is the corresponding change in SBH, *k* is the Boltzmann constant, and *T* = 300 K is the ambient temperature. Figure 3c plots the extracted ΔΦ as a function of bias for different incremental loads Δ*F*. The ΔΦ decreases with increasing bias and finally tends to zero when the applied bias gets close to the ideal SBH (∼ 0.9 eV) between GaN (the electron affinity of 3.6 eV for GaN [43]) and W (the work function of 4.47 eV for W [44]). Before saturation, ΔΦ increases with increasing Δ*F*.

For piezoelectric materials under a strain gradient, both piezoelectric and flexoelectric polarizations may in principle be generated. However, because reliable flexoelectric coefficients of GaN are not available, the calculation using reported Si coefficients is used only as an order‐of‐magnitude reference rather than a rigorous quantitative estimation. Under the macroscale tip‐loading condition, the generated strain gradient is relatively small compared with that produced by nanoscale AFM tips, and thus is insufficient to induce an appreciable flexoelectric polarization. The estimated flexoelectric polarization and charge density are much smaller than the piezoelectric components, indicating a negligible contribution from flexoelectricity (see Figure S6). This conclusion is further supported by the control experiment on a non‐piezoelectric n‐Si device under the same W‐tip loading configuration, which shows no comparable load‐dependent rectifying response (see Figure S7). These results confirm that the large electrical response in n‐GaN is dominated by strain‐gradient‐induced piezoelectric polarization rather than flexoelectricity.

The bias‐dependent ΔΦ for SGPT arises from the synergy of the interface polar symmetry and shielding effect of bulk piezo‐charges, as illustrated in Figure 3d. For a given tip load at low bias, the surface polar symmetry is strong due to a large interfacial electric field (Figure 3d‐i). This field repels free electrons away from the interface where bulk piezo‐charges are exactly located. Therefore, the shielding effect is weak, causing a pronounced reduction in the SBH. As the bias increases (Figure 3d‐ii), the polar region shrinks, and the portion of bulk piezo‐charges is out of the polar region and thus shielded by free electrons. As a result, the barrier is less modulated for SGPT. When the bias is large enough to vanish the interfacial electric field (Figure 3d‐iii), the piezo‐charges are fully shielded, with the SBH insensitive to load.

In conventional piezotronic devices, the piezo‐charges induced by uniform strain are mainly accumulated at the semiconductor interface. The free carriers can feel the polarization field induced by interface piezo‐charges, and yield a shielding effect. However, interface piezo‐charges are spatially separated from mobile carriers by the depletion region. Direct charge compensation is thus suppressed, and the barrier modulation by interface piezo‐charges is less sensitive to bias‐controlled shielding (see Figure S8). By contrast, in the strain‐gradient piezotronic device, the nonuniform strain induces bulk piezo‐charges distributed inside the GaN beneath the tip contact. These bulk piezo‐charges can overlap with free carriers when they are outside the electron‐depleted polar region, making their shielding degree tunable by the bias‐dependent interfacial electric field.

### 2.5 Size Effect on Piezotronic Modulation

**FIGURE 4:**
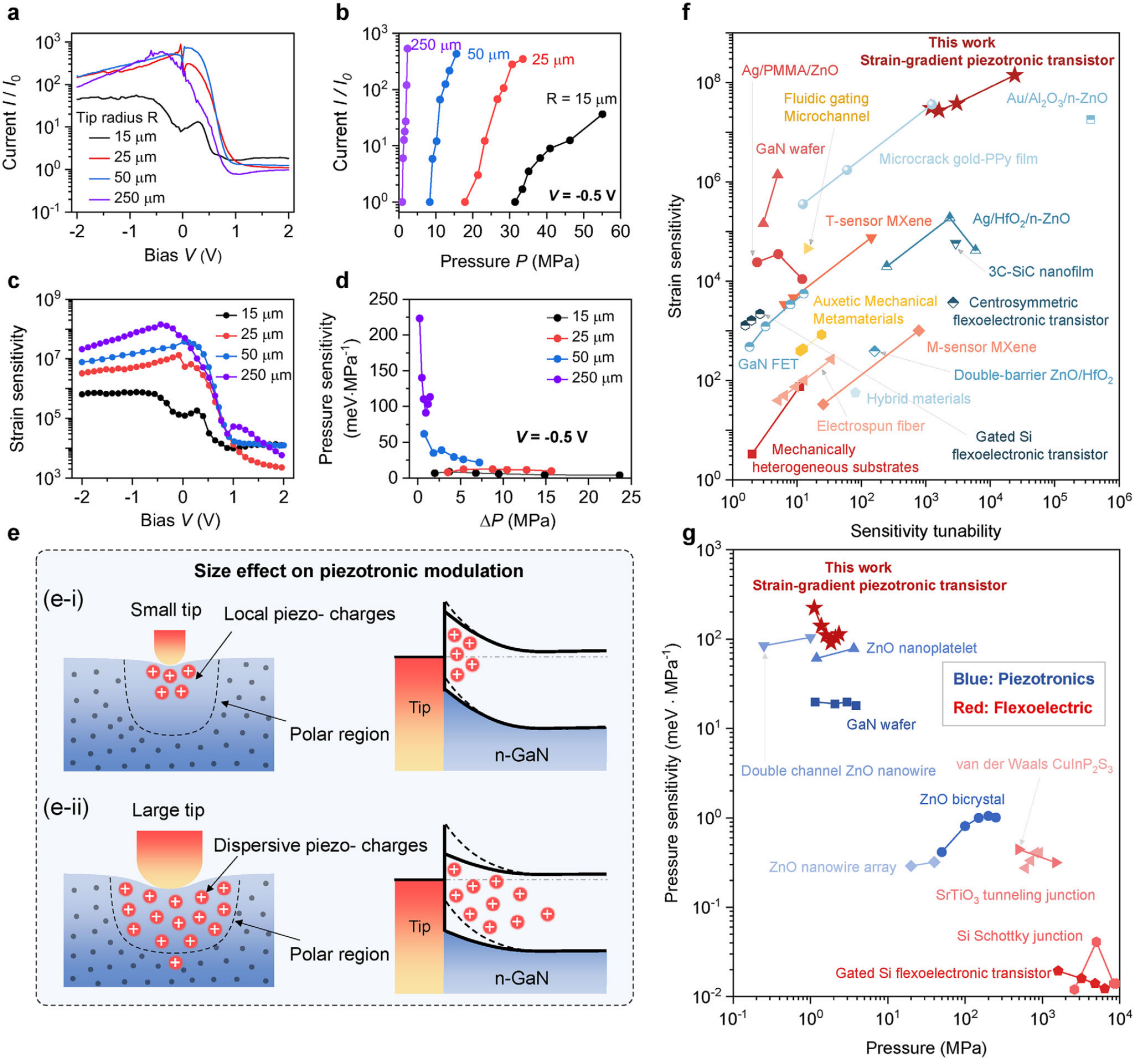
Size‐dependent piezotronic modulation in SGPT and performance comparison. (a) Bias‐dependent ΔI/I for different tip radius: R = 15 µm (F = 38.9 mN), 25 µm (F = 55.9 mN), 50 µm (F = 122.2 mN), and 250 µm (F = 459.8 mN). (b) ΔI/I as a function of pressure at bias *V* = −0.5 V. (c) Bias‐dependent strain sensitivity with the same tip radius and load as in (a). (d) Pressure sensitivity as a function of pressure variation ΔP at *V* = −0.5 V. (e) Schematic illustration of size effect on piezotronic modulation. For small‐radius tip (e‐i), the piezo‐charges are highly localized beneath the contact, whereas they are dispersive throughout the entire polar region for large‐radius tip (e‐ii). The latter causes a sufficient occupation of polar region and thus yields an evident band modulation. (f) Comparison of strain sensitivity and tunability for various strain sensors. (g) Comparison of pressure sensitivity and range for different pressure sensors.

Tip‐induced strain gradients exhibit a pronounced size dependence [34] (see Figure 1d), which would influence the piezotronic modulation. To evaluate it, we measure the I‐V characteristics of spherical‐indenter SGPT with radius R = 15, 25, and 250 µm (see Figure S9). Similar to the case of R = 50 µm, all devices exhibit the bias‐dependent high and low sensitivity regimes. Such bias dependence can be observed from the current change I/I_0_, as shown for a typical load of each tip in Figure 4a. Here, the current I_0_ corresponds to the initial load of 22.2, 35.1, 65.6 and 179.1 mN for R = 15, 25,50 and 250 µm, respectively, while I corresponds to loads of 38.9, 55.9, 122.2 and 459.8 mN. Therefore, the electrically tunable piezotronic modulation is a general phenomenon across tip sizes. Note that within the applied load ranges, there is no significant deformation occurred in all the tip indenters (as shown in Figure S10).

To analyze the sensing performance, Figure 4b plots I/I_0_ as a function of pressure at bias *V* = −0.5 V. The pressure is an averaged value, and similar to flexoelectric transistors [4, 8], it can be expressed as *P*  = *F*/2π*R* ^2^ . Because *P* scales inversely with *R* ^2^, the applied pressure on tips decreases with increasing radius. Meanwhile, as the tip radius increases, I/I_0_ rises steeply with *P*, causing an enhanced sensitivity for larger tip. This can be observed from the bias‐dependent strain sensitivity in Figure 4c. As expected, in the high‐sensitivity regime, the strain sensitivity increases with tip radius. Particularly at R = 250 µm, the device exhibits a maximum strain sensitivity of 1.43 × 10^8^ and a tunability of 24 403. We plot the pressure sensitivity (= ΔΦ/ΔP) as a function of its variation ΔP = P−P_0_ in Figure 4d for a bias *V* = −0.5 V. It shows that the pressure sensitivity also increases with tip radius. For R = 250 µm, the maximum pressure sensitivity reaches 223.2 meV·MPa^−1^.

A possible explanation for this size‐enhanced sensing performance is the scaling behavior of the strain field. Figure 4e shows the spatial distribution of bulk piezo‐charges and the resulting band modulation for different tip sizes. As demonstrated above, the bulk piezo‐charges are poorly shielded in the surface polar region due to the interfacial electric field that repels free electrons. Therefore, the bulk piezo‐charges filling the polar region more efficiently enable a stronger piezotronic effect. For small‐radius tips, the induced piezo‐charges are highly localized beneath the tip (Figure 4e‐i), making large portions of the polar region unoccupied with low barrier reduction. By contrast, a large tip radius causes a wide contact area and spreads the strain gradient (or piezo‐charges) over a larger volume (Figure 4e‐ii). These piezo‐charges occupy most of the polar region and collectively reshape the band bending, giving rise to a pronounced reduction in the Schottky barrier. To further support this mechanism, we quantitatively compared the spatial overlap between the electron‐depleted polar region and the strain‐gradient‐induced piezo‐charge distribution for different tip radii (see Figure S11). The results show that although the depletion‐region depth is comparable for different tip sizes, the larger tip produces a broader piezo‐charge distribution and stronger band modulation, thereby enabling more effective occupation of the polar region and enhanced piezotronic response.

### 2.6 Performance Comparison of Strain‐Gradient Piezotronic Transistors

To benchmark the sensing performance, Figure 4f compares the strain sensitivity and tunability of various strain sensors (Table S2). Our n‐GaN SGPT exhibits a strain sensitivity far exceeding that of other sensing mechanisms, including piezoresistive [27, 28, 45, 46, 47, 48], flexoelectric [4, 8], and piezotronic transistors [7, 13, 14, 16, 17, 18, 49]. Although piezotronic sensors generally outperform resistive and capacitive sensors due to their strong electromechanical coupling, the strain sensitivity achieved in our SGPT (up to 1.43 × 10^8^) is even higher than that of tunneling‐based sensors in Au/Al2O3/n‐ZnO (1.80 × 10^7^) [11] and Ag/HfO2/n‐ZnO (1.88 × 10^5^) [7], as well as double‐interface piezoelectric response in GaN bicrystal transistors (1.38 × 10^6^) [16]. By contrast, the flexoelectric transistors based on centrosymmetric semiconductors exhibit very limited strain sensitivity (< 2650) [4, 8], nearly five orders of magnitude lower than that of our SGPT. Additionally, the tunability of 24 403 of SGPT is also higher than other piezotronic sensors (interface‐dominated polarization) based on conductivity transformation (3–5 in GaN wafer [16], and 1.8–12.7 in GaN field effect transistor [18]), surface states of tunneling junction (2.4–12 in Ag/PMMA/ZnO [14], and 247–5893 in Ag/HfO2/ZnO [7]), and flexoelectronic transistor (2–2650) [4, 8]. More importantly, this tunability can be controlled by the bias voltage, making it possible to achieve a continuous switching of high and low sensitivity. This offers an expedient strategy for integration of multiple sensing functions in a single sensor.

To further evaluate device‐to‐device reproducibility, we measured ten different SGPT devices and statistically summarized their strain sensitivity under different bias voltages (see Figure S12). The devices exhibit good consistency over the bias range from −2 to 1 V, with a similar trend that the strain sensitivity remains high under negative bias and decreases under positive bias. This reproducible bias‐dependent sensitivity confirms the robustness of the electrically tunable piezotronic response.

Figure 4g further compares the pressure sensitivity of various piezotronic [16, 22, 23, 24, 25, 50] and flexoelectric pressure sensors [4, 8, 30, 32]. Although both SGPT and flexoelectric transistors are strain‐gradient devices, the former achieves an ultrahigh pressure sensitivity of 223.2 meV·MPa^−1^. This sensitivity is four orders of magnitude higher than that of flexoelectric transistors (0.01–0.05 meV·MPa^−1^), in Si‐based Schottky [4, 8], SrTiO_3_ tunneling junction [30], and van der Waals CuInP_2_S_3_ [32]. Meanwhile, SGPTs are operated under pressure conditions (∼1.0 MPa) that are far lower than the nanoscale tip of an atomic force microscope (> 500 MPa). On the other hand, our SGPT also exhibits clear performance advantage compared with those piezotronic transistors based on uniform strain, such as ZnO and CdSe nanowire (0.3–104 meV·MPa^−1^) [22, 23, 25], ZnO nanoplatelet (61–78 meV·MPa^−1^) [24], and GaN wafer cases (18.1–19.8 meV·MPa^−1^) [16].

### 2.7 Dynamically Stable and Tunable Strain Sensors

**FIGURE 5:**
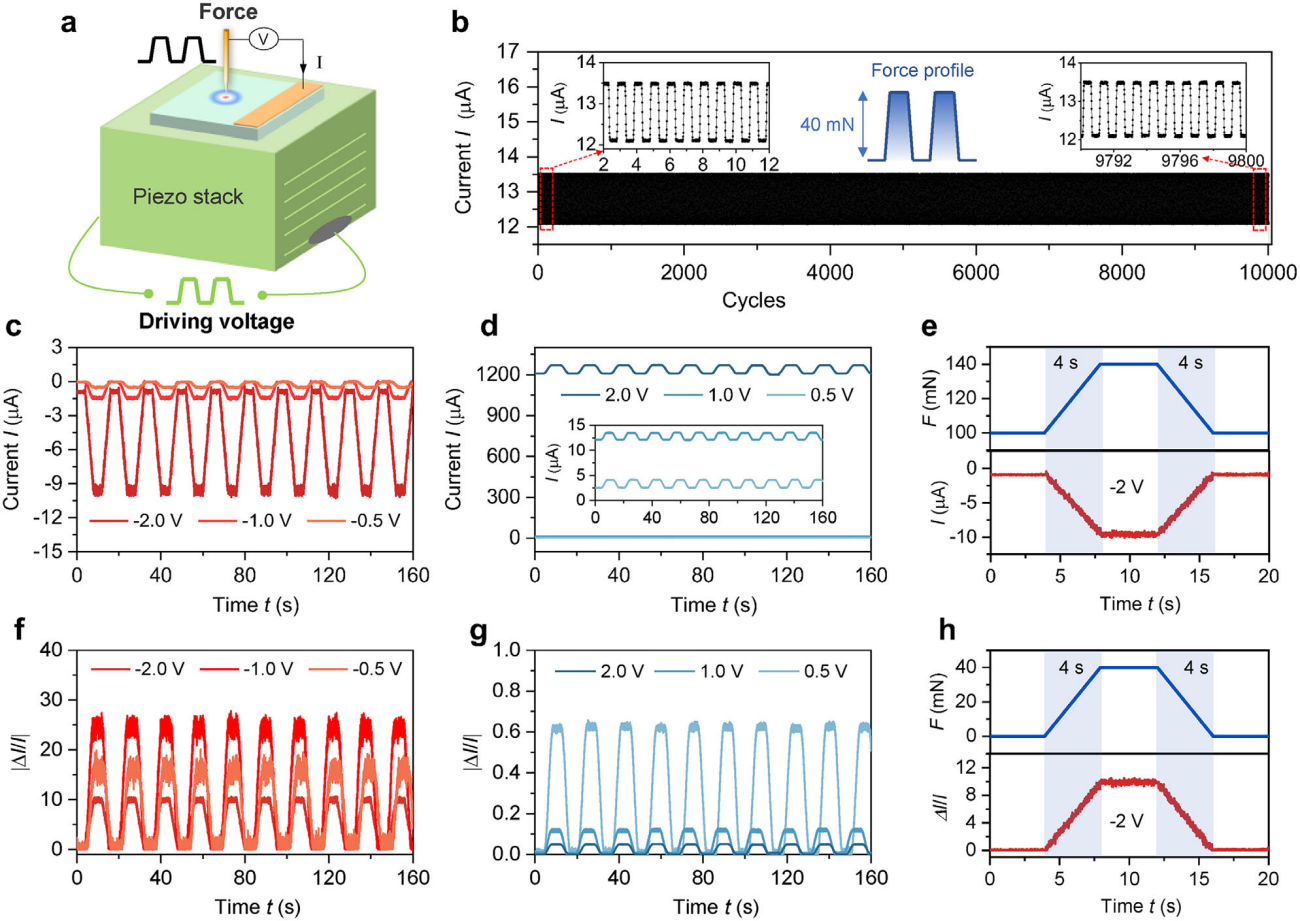
Dynamic testing of a highly stable and tunable‐sensitivity SGPT. (a) Dynamic testing setup with a square‐wave load generated by the driving voltage of a piezoelectric stack actuator. (b) Output current versus time (I‐t) over 10,000 repeated load cycles; the left and right insets show I–t traces near the start and end, respectively, and the middle inset shows the profile of the applied load (amplitude 40 mN). I–t traces over 10 cycles under reverse bias (c), and forward bias (d); inset in (d) shows I–t at *V* = 0.5 and 1.0 V. (e) The applied load (top) and current (bottom) at *V* = −2 V within a single cycle. Time evolution of the absolute current change |ΔI/I |under reverse bias (f), and forward bias (g). (h) The applied load (top) and |ΔI/I| (bottom) at *V* = −2 V within a single cycle.

To further assess device stability under dynamic loading, we monitor the time‐resolved current of SGPT (radius R = 50 µm) subjected on a periodic load. The dynamic measurement setup is schematically shown in Figure 5a, with a square‐wave load generated by a piezoelectric stack. The force waveforms are shaped with smooth ramp‐up and ramp‐down profiles to avoid an abrupt step that would introduce mechanical delay to perturb the electrical response [see Figure S13].

Figure 5b plots the output current at a fixed bias of 1.0 V under periodic load with an increment of 40 mN (see inset for force profile). We can see that the current remains remarkably stable for SGPT working continuously even over 10 000 cycles (10 s per cycle). The high‐ and low‐current do not show an evident drift at the beginning and end of the test (insets, Figure 5b). This demonstrates the excellent mechanical durability of our SGPT under dynamic conditions.

Next, we study the bias‐dependent sensitivity in dynamical condition. Figure 5c,d show the current response over ten load cycles under forward (0.5, 1.0, and 1.5 V) and reverse (−0.5, −1.0, and −1.5 V) bias, respectively. The temporal profile of the mechanical input matches well with the electrical response (Figure 5e at *V* = −2 V). Additionally, the current magnitude grows with |*V*| at both forward and reverse bias. The current spans multiple orders of magnitude as the forward bias is increased from 0.5 V to 2.0 V (inset, Figure 5d), whereas reverse bias produces lower current but a clearer, high‐sensitivity response (Figure 5c). Note that such bias‐dependent dynamical response is repeatable for tip positioning at different positions of GaN (see Figure S14).

To quantify sensing sensitivity, we obtain the absolute value of current change |ΔI/I| as a function of time. As we can see, the |ΔI/I| shows high bias dependence at both forward and reverse bias voltages (Figure 5f,g). However, the |ΔI/I| at reverse bias is far higher than that of the forward case. For example, at −2 V, |ΔI/I| reaches ∼10, which is roughly 200 times higher than the value at +2 V. The profile of the load and |ΔI/I| also coincides at a single cycle (Figure 5h at *V* = −2 V). These results suggest that even under dynamic loading, our SGPT maintains highly repeatable and tunable sensing performance.

For practical applications, the tip‐contact structure can be translated into a packaged pressure‐sensing architecture by replacing the tungsten tip with a robust protrusion, hemispherical bump, patterned rigid contact, or microstructured top electrode. A vertical stack containing a force‐collecting layer, fixed protrusion electrode, GaN Schottky sensing layer, and bottom Ohmic electrode could maintain stable contact under a small preload and generate a localized strain‐gradient field under external pressure. Such a design could be further extended to protrusion‐array‐based tactile pixels, where the operating bias switches each unit between high‐ and low‐sensitivity states. This provides a feasible route toward adaptive pressure sensors, intelligent tactile systems, robotic skins, and human–machine interfaces.

## 3 Discussion

In summary, we have demonstrated an SGPT based on a macroscale tip subjected to n‐GaN. Engineering interface polar symmetry enables an ultrasensitive and electrically tunable piezotronic response. By coupling the bias‐dependent polar symmetry and shielding effect of bulk piezo‐charges, the SGPT exhibits high and low sensitivity regimes that can be continuously tuned by the bias voltage. This mechanism is universal across different indenter geometries and radii, with spherical tips delivering the stronger piezotronic response. The SGPT sensor achieves a wide tunability of 24 403, a large pressure sensitivity of 223.2 meV·MPa^−1^, and an ultrahigh strain sensitivity of 1.43 × 10^8^, outperforming state‐of‐the‐art Si‐based flexoelectronic transistors by several orders of magnitude. In addition, increasing the tip radius enables enhanced sensing performance due to the size effect of the strain gradient. Our SGPT also exhibits excellent mechanical durability over 10 000 loading cycles and maintains electrically tunable sensitivity under dynamic load. These results demonstrate the combination of strain gradient and symmetry engineering as a promising strategy to achieve tunable piezotronics, with great potential for the integration of more sensing functionalities.

## 4 Methods

### 4.1 n‐GaN Wafer Materials

Commercial (0001)‐oriented n‐type GaN wafers (Suzhou Nanowin Science and Technology Co., Ltd.) were grown by metalorganic chemical vapor deposition (MOCVD) on *c*‐plane sapphire. The wafer had a diameter of 100 ± 0.2 mm and contained a 4.5±0.5 µm Si‐doped GaN epilayer with a Ga‐terminated (0001) surface (*c*‐axis up; miscut 0.2 ± 0.1°). At 300 K, the GaN layer was n‐type with resistivity >0.5 Ω·cm, mobility > 300 cm^2^/V·s and electron concentration < 2 × 10^17^ cm^−^^3^. The threading dislocation density was <5 × 10^8^ cm^−^^2^. The GaN‐on‐sapphire substrates were supplied in standard single‐side polished form, and the GaN surface roughness was < 0.3 nm. The n‐GaN wafers were sequentially cleaned by acetone, isopropyl alcohol, and deionized water for 10 min each in an ultrasonic bath, followed by 80‐s oxygen plasma treatment. Then, the cleaned n‐GaN deposits Al electrodes within 5 min via magnetron sputtering to form Ohmic contacts.

### 4.2 The Application of Tip Force

Both static and dynamic loads are applied to n‐GaN using a home‐made tip‐loading stage (see Figure S3). Here, commercial tungsten conductive tips (radius 15–250 µm) were first bonded to an insulating mount and mechanically clamped beneath a movable plunger. The opposite end of the plunger was coupled to a vertically adjustable nut through a compliant spring. The spring buffers the tip motion and prevents tip deformation at contact with the sample. Rotating the nut compressed the spring and generated a controllable static normal force on the tip, which was transferred to the sample surface. A high‐sensitivity force sensor placed beneath the sample was used to directly measure the tip load. In addition, for dynamic loading, a piezoelectric stack actuator (PSt150/10 × 10/20L, E53.C1K‐H, CoreMorrow) was inserted beneath the sample and driven electrically to produce controlled periodic displacement. This approach can provide large tip forces with precise and long‐duration electrical tunability.

### 4.3 Electrical Transport Measurements Under Tip Load

The electrical transport of n‐GaN under tip loading was measured by using a dedicated setup consisting of source meter (Keithley 2614B), low‐noise current preamplifier (SR570, Stanford Research Systems), a piezoelectric stack, and a digital oscilloscope (Tektronix TBS1000X Series). The Keithley 2614B digital source meter was used to support voltage signal and simultaneously detect the current. The source meter was connected between the Al contact on n‐GaN and the metallic tip. Once the tip was brought into contact under a sufficient initial load, which increases with tip radius, the device exhibited stable and reproducible *I–V* characteristics. However, the applied load cannot be increased arbitrarily, otherwise excessive force damages the tip apex and degrades device performance. The *I–V* characteristics are acquired over −2 to +2 V in 20 mV steps. For time‐resolved measurements under periodic loading, the output current was recorded by the SR570 in combination with the digital oscilloscope.

### 4.4 The Performance of Tip‐Based Piezotronics Sensors

All measurements were performed in air at room temperature. Similar to tip‐based flexoelectronic sensors [4, 8], strain sensitivity is defined as $\mathrm{SS}\;=\frac{I-I_{0}}{I_{0}\Delta \varepsilon }$, with $\Delta \varepsilon =\frac{\Delta F}{\pi R^{2}E}$, the zero‐load current *I*_0_, the changed load Δ*F*, and the Young modulus of GaN *E* = 352 GPa. It should be noted that the definitions of strain sensitivity and tunability vary among different types of strain sensors in the literature. For example, piezoresistive sensors are commonly evaluated by gauge factor, piezotronic or tunneling‐based sensors often use relative current change normalized by strain, and electrically switchable devices may define tunability as the ratio between high‐ and low‐sensitivity states. In this work, the strain sensitivity is defined as the relative current change normalized by the averaged strain increment induced by tip loading, and the tunability is defined as the ratio between the maximum and minimum strain sensitivity within the applied bias window. Therefore, the comparison in Figure 4f and Table S2 should be regarded as a general benchmark of sensing performance rather than a strictly identical‐definition ranking.

## Author Contributions

**Gongwei Hu**: conceptualization, validation, methodology, writing – review and editing, writing – original draft, supervision, funding acquisition. **Yong Chao**: methodology, software. **Xiaoming Dong**: methodology, data curation, investigation, writing – original draft. **Min Liu**: data curation. **Liqing Pan**: data curation. **Menglu Li**: software. **Fobao Huang**: supervision, validation, methodology, writing – original draft. **Lijie Li**: writing – review and editing, supervision, funding acquisition. **Yihan Zhang**: methodology, software. **Wei Huang**: supervision.

## Conflicts of Interest

The authors declare no conflict of interest.

## Supporting information




**Supporting File**: advs76341‐sup‐0001‐SuppMat.pdf.

## Data Availability

The data that support the findings of this study are available from the corresponding author upon reasonable request.
