# Supplementary material for: Electrically Tunable Piezotronic Transistor by Coupling Interface Polar Symmetry and Strain Gradient
Source: Adv Sci (Weinh). 2026 Jul 3;13(53):e76341. doi: 10.1002/advs.76341 (PMC13334598; doi:10.1002/advs.76341)
Supplement: Supplementary file 1 [file ADVS-13-e76341-s001.pdf]

Supplemental Materials

Electrically Tunable Piezotronic Transistor by Coupling Interface Polar Symmetry and Strain Gradient

Gongwei Hu[1,*], Xiaoming Dong[1], Yihan Zhang[1], Yong Chao[2,3,4], Menglu Li[1], Min Liu[1], Liqing Pan[1], Lijie Li[5,*], and Wei Huang[2,*], Fobao Huang[2,3,4,*]

1 *Hubei Engineering Research Center of Weak Magnetic-field Detection, College of Mathematics and Physics, China Three Gorges University, Yichang 443002, China*

2 *State Key Laboratory of Flexible Electronics (LoFE) & School of Integrated Circuits (School of Microelectronics), Northwestern Polytechnical University, Xi'an 710072, China*

3 *Shenzhen Research Insititute of Northwestern Polytechnical University, Shenzhen 518057, China*

4 *Yangtze River Delta Research Institute of Northwestern Polytechnical University, Taicang 215400, China*

5 *College of Engineering, Swansea University, Swansea SA1 8EN, UK*

**Corresponding authors*: E-mail: gwhu@ctgu.edu.cn; L.Li@swansea.ac.uk; fbhuang@nwpu.edu.cn; iamwhuang@nwpu.edu.cn

Table of contents

Note S1: Spherical indentation strain field and piezoelectric effect.......... 2
Note S2: The averaged strain gradient in a spherical indenter 5
Figure S1. The strain gradient effect on GaN.......... 8
Figure S2. Size scaling law for a tip-induced strain and strain gradient. 9
Figure S3. The home-made tip-loading stage for measurement.......... 10
Figure S4. The contact types of n-GaN device.......... 10
Figure S5. Reversibility of bias-dependent sensitivity switching in SGPT.......... 11
Figure S6. The comparison of flexoelectricity and piezoelectricity in GaN film under a tip load. 12
Figure S7. The comparison of strain-gradient flexoelectronic and piezotronic effect. 13
Figure S8. Schematic comparison of shielding behaviors between interface and bulk piezo-charges.......... 14
Figure S9. The size effect on the strain-gradient piezotronic transistors. 15
Figure S10. The optical microscope photographs of spherical tungsten tips before and after load. 16
Figure S11. Quantitative comparison of the depletion region and piezo-charge distribution for different tip radii.......... 17
Figure S12. Device-to-device reproducibility of strain-gradient piezotronics transistor. 18

Figure S13. The impact of force waveform profile on dynamic test for strain-gradient piezotronic transistors.......... 19
Figure S14. The repeatability of dynamic response for strain-gradient piezotronic transistors. 20
Reference.......... 21

Note S1: Spherical indentation strain field and piezoelectric effect

The gradient strain is generated by the tip force applied during mechanical loading, and the stress field is obtained using Hertzian contact theory [1]. For a spherical indenter, the stress distribution can be expressed in cylindrical coordinates as follows

$$\frac{\sigma_r}{p_m}=\frac{3}{2}\left\{\begin{array}{l}\frac{1-2v}{3}\frac{a^2}{r^2}\left[1-\left(\frac{z}{u^{1/2}}\right)^3\right]+\left(\frac{z}{u^{1/2}}\right)^3\frac{a^2u}{u^2+a^2z^2}\\+\frac{z}{u^{1/2}}\left[\frac{u(1-v)}{a^2+u}+\frac{u^{1/2}(1+v)}{a}\tan^{-1}\left(\frac{a}{u^{1/2}}\right)-2\right]\end{array}\right\} \tag{S1}$$

$$\frac{\sigma_\theta}{p_m}=-\frac{3}{2}\left\{\frac{1-2v}{3}\frac{a^2}{r^2}\left[1-\left(\frac{z}{u^{1/2}}\right)^3\right]+\frac{z}{u^{1/2}}\left[2v+\frac{u(1-v)}{a^2+u}-\frac{u^{1/2}(1+v)}{a}\tan^{-1}\left(\frac{a}{u^{1/2}}\right)\right]\right\} \tag{S2}$$

$$\frac{\sigma_z}{p_m}=-\frac{3}{2}\left(\frac{z}{u^{1/2}}\right)^3\left(\frac{a^2u}{u^2+a^2z^2}\right),\quad \frac{\tau_{rz}}{p_m}=-\frac{3}{2}\left(\frac{rz^2}{u^2+a^2z^2}\right)\left(\frac{a^2u^{1/2}}{a^2+u}\right) \tag{S3}$$

$$u=\frac{1}{2}\left[\left(r^2+z^2-a^2\right)+\sqrt{\left(r^2+z^2-a^2\right)^2+4a^2z^2}\right] \tag{S4}$$

Here, $p_m = F/\pi a^2$ is the mean stress, and $a=\left(\frac{3FR}{4E}\right)^{1/3}$ is the deformation radius, E is the Young modulus, v is the Poisson ratio, R is the indenter radius, $r=\sqrt{x^2+y^2}$ and z are cylindrical coordinates. The radial symmetry in spherical indenter leads to θ-independent radial stress σ_r, loop stress σ_θ, normal stress σ_z, and shear stress τ_{rz}.

The stress field generated by a flat cylindrical indenter pressing into a planar surface resembles that of the classical Hertzian contact. In many respects, the geometry of a flat punch offers advantages over a spherical indenter, as the contact radius remains constant and independent of the applied load. This eliminates one of the key load-dependent variables and simplifies the mechanical analysis. The stress field can be calculated from

$$\frac{\sigma_r}{p_m} = -\frac{1}{2}\left[J_1^0 - \frac{z}{a}J_2^0 - (1-2v)\frac{a}{r}J_0^1 + \frac{z}{r}J_1^1\right]$$
$$\frac{\sigma_\theta}{p_m} = -\frac{1}{2}\left[2vJ_1^0 + (1-2v)\frac{a}{r}J_0^1 - \frac{z}{r}J_1^1\right] \tag{S5}$$
$$\frac{\sigma_z}{p_m} = -\frac{1}{2}\left(J_1^0 + \frac{z}{a}J_2^0\right), \quad \frac{\tau_{rz}}{p_m} = -\frac{1}{2}\frac{z}{a}J_2^1$$

Here, the parameters of J_1^0, J_0^1, J_2^1, J_2^0, J_1^1 are given by

$$J_1^0 = R^{-1/2}\sin\frac{\phi}{2}, \quad J_0^1 = \frac{a}{r}\left(1 - R^{1/2}\sin\frac{\phi}{2}\right), \quad J_2^1 = \frac{r}{a}R^{-3/2}\sin\frac{3\phi}{2}$$
$$J_2^0 = \left(1+\frac{z^2}{a^2}\right)^{1/2} R^{-3/2}\sin\left(\frac{3\phi}{2} - \theta\right), \quad J_1^1 = \left(1+\frac{z^2}{a^2}\right)^{1/2}\frac{a}{r}R^{-1/2}\sin\left(\theta - \frac{\phi}{2}\right) \tag{S6}$$
$$R = \left[\left(\frac{r^2}{a^2} + \frac{z^2}{a^2} - 1\right)^2 + \frac{4z^2}{a^2}\right]^{1/2}, \quad \tan\phi = \frac{2z}{a}\left(\frac{r^2}{a^2} + \frac{z^2}{a^2} - 1\right)^{-1}, \quad \tan\theta = \frac{a}{z}$$

In the Cartesian coordinates, all the shear stress is obtained by replacing r with x or y in τ_{rz}. Axis stresses in *x*-*y* plane are obtained by the matrix transformation $U = \begin{pmatrix} cos\theta & -sin\theta \\ sin\theta & cos\theta \end{pmatrix}$ on σ_r and σ_θ. Then, the strain is given by the Hooke's law

$$\varepsilon_{ii} = \frac{1}{E}\left[\sigma_{ii} - v\left(\sigma_{jj} + \sigma_{kk}\right)\right], \quad \varepsilon_{ij} = \frac{(1+v)\sigma_{ij}}{E} \tag{S5}$$

Here, *i*, *j*, *k* stand for the *x*, *y*, *z* axis with different values. Once the strain is obtained, we can calculate the strain gradient

$$\varepsilon_{ij,k} = \frac{\partial \varepsilon_{ij}}{\partial x_k} \tag{S6}$$

For a piezoelectric semiconductor based on wurtzite GaN, the piezoelectric polarization can be calculated by

$$\begin{pmatrix} p_x \\ p_y \\ p_z \end{pmatrix} = \begin{pmatrix} 0 & 0 & 0 & 0 & d_{15} & 0 \\ 0 & 0 & 0 & d_{15} & 0 & 0 \\ d_{31} & d_{31} & d_{33} & 0 & 0 & 0 \end{pmatrix} \begin{pmatrix} \sigma_{xx} \\ \sigma_{yy} \\ \sigma_{zz} \\ \sigma_{yz} \\ \sigma_{xz} \\ \sigma_{xy} \end{pmatrix} \tag{S7}$$

where d_{31} d_{33}, and d_{15} are piezoelectric coefficient. The piezo-charges can be given by

$$\rho_{piezo} = -\nabla \cdot \boldsymbol{P} = -\left(\frac{\partial p_x}{\partial x} + \frac{\partial p_y}{\partial y} + \frac{\partial p_z}{\partial z}\right) \tag{S8}$$

Here, the piezoelectric polarization is a vector $\boldsymbol{P} = p_x\hat{\boldsymbol{x}} + p_y\hat{\boldsymbol{y}} + p_z\hat{\boldsymbol{z}}$. Because the piezo-

charges are the gradient of piezoelectric polarization, and thus relevant with the strain gradient.

For comparison with flexoelectric effect, we also study the flexoelectric polarization of GaN. However, due to the absence of relevant reports of flexoelectric coefficients for GaN, we replace them with those of Si for a simple understanding.

Due to the relationship $\varepsilon_{ij} = \varepsilon_{ji}$, the total number of flexoelectric coefficients are 36. However, the high crystal symmetry o_h in Si leads to only three independent components in flexoelectric tensor: $\mu_{1111} = \mu_{2222} = \mu_{3333} = \mu_{11}$, $\mu_{1133} = \mu_{2233} = \mu_{1122} = \mu_{2121} = \mu_{3232} = \mu_{3131} = \mu_{111}$, $\mu_{1221} = \mu_{1331} = \mu_{2112} = \mu_{2332} = \mu_{3223} = \mu_{3113} = \mu_{14}$ [2]. Therefore, the matrix expression of flexoelectric tensor can be written as

$$\mu = \begin{pmatrix} \mu_{11} & \mu_{111} & \mu_{111} & 0 & 0 & 0 \\ \mu_{111} & \mu_{11} & \mu_{111} & 0 & 0 & 0 \\ \mu_{111} & \mu_{111} & \mu_{11} & 0 & 0 & 0 \\ 0 & 0 & 0 & \mu_{14} & 0 & 0 \\ 0 & 0 & 0 & 0 & \mu_{14} & 0 \\ 0 & 0 & 0 & 0 & 0 & \mu_{14} \end{pmatrix} \tag{S8}$$

The flexoelectric polarization can be obtained [3]

$$P_i = \mu_{ijkl} \frac{\partial \varepsilon_{jk}}{\partial x_l} \tag{S9}$$

with $x_l = (x, y, z)$. Combining the flexoelectric polarization equation, the cubic symmetry is used to reduce P_r to [4]

$$P_r = \mu_{11}\varepsilon_{rr,r} + \mu_{111}\varepsilon_{rz,z} + \mu_{14}\varepsilon_{\theta\theta,r} + \mu_{111}\varepsilon_{zr,z} + \mu_{14}\varepsilon_{zz,r} \tag{S10}$$

We can see from (S10) that flexoelectric coefficient μ_{11}, μ_{14}, and μ_{111} corresponds to longitudinal, transverse and shear strain gradient, respectively. There is a further relationship for three non-zero flexoelectric coefficients $\mu_{14} = (\mu_{11} - \mu_{111})/2$. We can further derive

$$P_r = \mu_{11}\varepsilon_{rr,r} + \mu_{14}(\varepsilon_{\theta\theta,r} + \varepsilon_{zz,r}) + 2\mu_{111}\varepsilon_{rz,z} \tag{S11}$$

$$P_z = \mu_{11}\varepsilon_{zz,z} + \mu_{14}(\varepsilon_{\theta\theta,z} + \varepsilon_{rr,z}) + 2\mu_{111}\varepsilon_{rz,r}, \quad P_\theta = 0 \tag{S12}$$

The flexoelectric polarization charges can also be attained from $\rho_{flexo} = -\nabla \cdot \boldsymbol{P}_{flexo}$. Table S1 lists the material parameters for wurtzite GaN and Si. Here, we do not derive their explicit expressions due to the complexity, and instead, they are attained by numerical calculation.

Table S1. The basic parameters for wurtzite piezoelectric semiconductor GaN and Si. The relevant parameters are found in Refs. [5-8]

Parameters	GaN	Si
Young's modulus (GPa)	352	170
Poisson's ratio	0.20	0.28
d_{31} (pm/V)	-1.6	--
d_{33} (pm/V)	3.1	--
d_{15} (pm/V)	3.1	--
μ_{11} (nC/m)	--	1.3
μ_{111} (nC/m)	--	0.4
μ_{14} (nC/m)	--	0.4

Note S2: The averaged strain gradient in a spherical indenter

To quantify the overall strength of the strain-gradient field induced by spherical indentation, we compute an averaged strain gradient over the mechanically deformed region. Based on the Hertzian stress solution [1], the full strain tensor is obtained through isotropic elasticity. According to Ref [4], an effective strain-gradient can be defined as

$$\langle\nabla\varepsilon\rangle_{eff} = 3\varepsilon_{zz,z} + 2\varepsilon_{xz,x} + 2\varepsilon_{yz,y} + \varepsilon_{xx,z} + \varepsilon_{yy,z} \tag{S9}$$

which captures the contribution associated with the out-of-plane gradients of the principal strain components. Since $(\nabla\varepsilon)_{eff}$ varies significantly within the indented volume, we characterize its global strength by spatial averaging

$$\langle\nabla\varepsilon\rangle = \frac{1}{V_{def}}\iiint\langle\nabla\varepsilon\rangle_{eff}dV \tag{S10}$$

where $V_{def} = a^3$ denotes the characteristic deformation volume defined by the contact radius a. This averaging approach removes the dominance of localized peak gradients and reveals a clear size dependence governed primarily by the indenter radius. Because the piezo-charge induced by strain gradients scales linearly with $(\nabla\varepsilon)_{eff}$, the averaged quantity $\langle\nabla\varepsilon\rangle$ provides a physically meaningful measure for the comparison of different tip sizes and for assessing their electromechanical impact. According to averaged definition, we can give the size sensitivity of strain gradient as $\langle\frac{\partial\nabla\varepsilon}{\partial R}\rangle$ with tip radius R.

Table S2. The comparison of strain sensitivity and tunability for various strain sensors.

Material	Strain sensitivity	Tunability	Ref
Au/Al2O3/n-ZnO	1.8×10^{7}	3.6×10^{5}	[9]
mechanically heterogeneous substrates	3.3 ~ 75.8	2 ~ 11	[10]
Ag/PMMA/ZnO	1.1×10^{4} ~ 2.4×10^{4}	2.4 ~ 12	[11]
Back-to-back GaN wafer	1.45×10^{5} ~ 1.38×10^{6}	3 ~ 5	[8]
T-sensor MXene	3380 ~ 75000	6.4 ~ 142	[12]
M-sensor MXene	33 ~ 1010	25.4 ~ 777	[12]
Electrospun fiber	39.6 ~ 266.6	5.1 ~ 34	[13]
fluidic gatin Microchannel	45300	14.5	[14]
Auxetic Mechanical Metamaterials	393 ~ 835	11.2 ~ 23.9	[15]
Hybrid materials	56.64	80.6	[16]
Microcrack gold-Ppy film	3.56×10^{5} ~ 3.6×10^{7}	12.3 ~ 1.24×10^{3}	[17]
GaN FET	1330	4.2	[18]
Double-barrier ZnO/HfO2	390	160	[19]
Ag/HfO2/n-ZnO	503 ~ 41413	6.3 ~ 5892.7	[20]
3C-SiC nanofilm	58000	2900	[21]
Centrosymmetric flexoelectronic transistor	2650	3700	[22]
flexoelectronic transistor	1288 ~ 2189	1.57 ~ 2.66	[23]
Tip-based GaN	3.8×10^{7} ~ 1.4×10^{8}	3031 ~ 24000	This work

Table S3. The comparison of pressure sensitivity and range for various pressure sensors.

Type	Material	Pressure (MPa)	Pressure sensitivity	Ref
Piezotronic	GaN wafer	1.15 ~ 3.01	18.06 ~ 19.83	[8]
	ZnO Bicrystal	50 ~ 250	0.414 ~ 1.018	[24]
	ZnO Nanoplatelet	1.2 ~ 3.64	60.97 ~ 78.23	[25]
	Double Channel ZnO	0.25 ~ 1.0	84.2 ~ 104.4	[26]
	ZnO nanowire array	20 ~ 40	0.29 ~ 0.32	[27]
	CdSe nanowire	113	0.69	[28]
Flexoelectric	Gated Si flexoelectronics	1600 ~ 6400	0.0123 ~ 0.0194	[23]
	Si Schottky junction	2600 ~ 9068	0.012 ~ 0.014	[22]
	SrTiO3 tunneling junction	601.8 ~ 920.4	0.27 ~ 0.41	[29]
	van der Waals $CuInP_2S_6$	495.6 ~ 1486.8	0.32 ~ 0.44	[30]
	n-GaN strain gradient piezotronic transistor	0.91 ~2.3	223.2 ~ 113. 4	This work

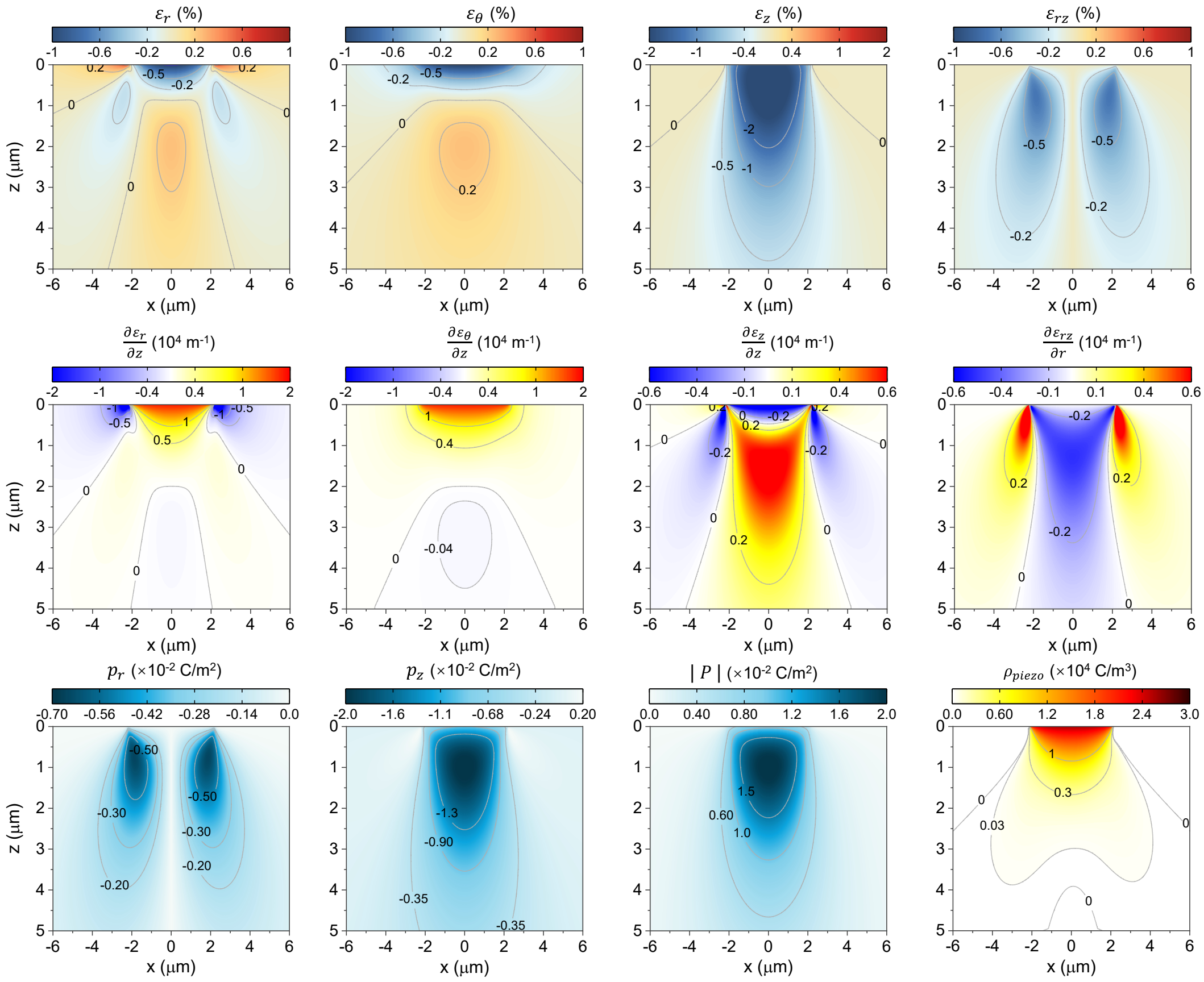

Figure S1. The strain gradient effect on GaN. The profile of strain ($\boldsymbol{\varepsilon_r}, \boldsymbol{\varepsilon_\theta}, \boldsymbol{\varepsilon_z}$, and $\boldsymbol{\varepsilon_{rz}}$), strain gradient ($\boldsymbol{\partial\varepsilon_r/\partial z}, \boldsymbol{\partial\varepsilon_\theta/z}, \boldsymbol{\partial\varepsilon_z/\partial z}$, and $\boldsymbol{\partial\varepsilon_{rz}/\partial r}$), piezoelectric polarization ($\boldsymbol{p_r}, \boldsymbol{p_z}, |\boldsymbol{P}|$), and piezo-charge ($\boldsymbol{\rho_{piezo}}$) for a spherical indenter with radius R = 50 μm and load F = 100 mN.

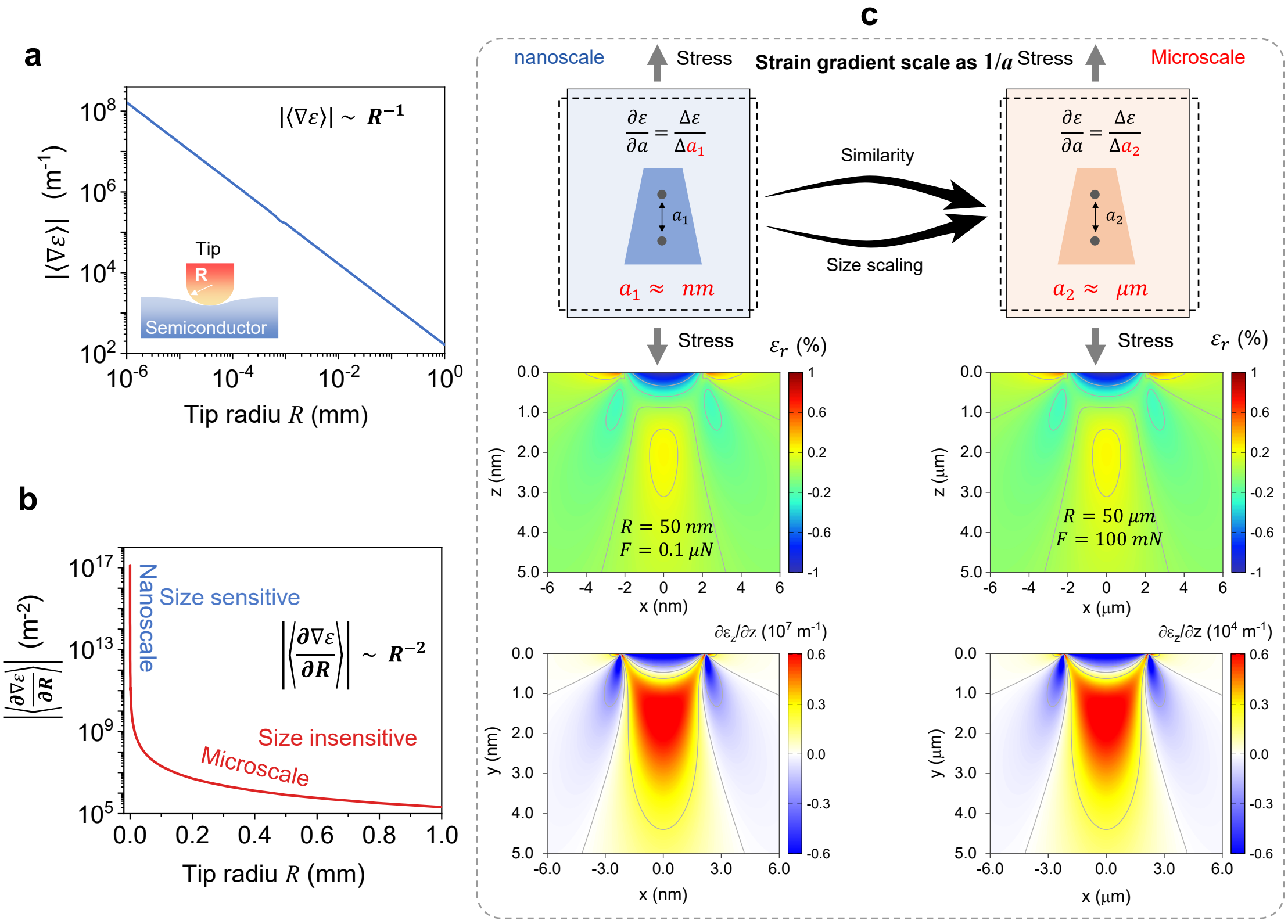

Figure S2. Size scaling law for a tip-induced strain and strain gradient. The absolute value of average strain gradient $|\langle\nabla\boldsymbol{\varepsilon}\rangle|$ (a) and strain-gradient sensitivity $|\langle\partial\nabla\boldsymbol{\varepsilon}/\partial\boldsymbol{R}\rangle|$ (b) as a function of tip radius R. The strain gradient and its sensitivity meet the law of linear ($|\langle\nabla\boldsymbol{\varepsilon}\rangle|\sim\boldsymbol{R}^{-1}$) and square scaling ($|\langle\partial\nabla\boldsymbol{\varepsilon}/\partial\boldsymbol{R}\rangle|\sim\boldsymbol{R}^{-2}$). It shows in (b) that, $|\langle\partial\nabla\boldsymbol{\varepsilon}/\partial\boldsymbol{R}\rangle|$ is extremely high ($10^{13} \sim 10^{17}$ m^{-2}) at nanoscale, whereas it becomes very low ($10^6 \sim 10^9$ m^{-2}) at microscale. This means that strain gradient generated by a nanoscale tip is much more sensitive to size than that of the microscale tip. Such liner scaling law of strain gradient can be explained from (c). Under the same far-field stress (upper plane), two triangular inclusions with identical aspect ratios but different absolute sizes will exhibit strain gradients that scale inversely with their characteristic length a_1, i.e., proportional to $1/a_1$. To verify that this scaling behavior also applies to tip case, we calculated the strain and strain-gradient fields for two spherical indenters with radius of 50 nm (load: 0.1 μN) and 50 μm (load: 100 mN), respectively. The strain maps (mid plane) show that the two systems exhibit completely identical strain magnitudes and spatial distributions, confirming that the strain field itself is scale-invariant when the geometry and aspect ratio are preserved. In contrast, the corresponding strain-gradient fields, while sharing the same spatial profile, differ in magnitude by three orders of magnitude, the same ratio as the tip radius. This result demonstrates that the strain gradient scales inversely with the characteristic tip size, fully consistent with the $1/a_1$ scaling behavior predicted for geometrically similar inclusions.

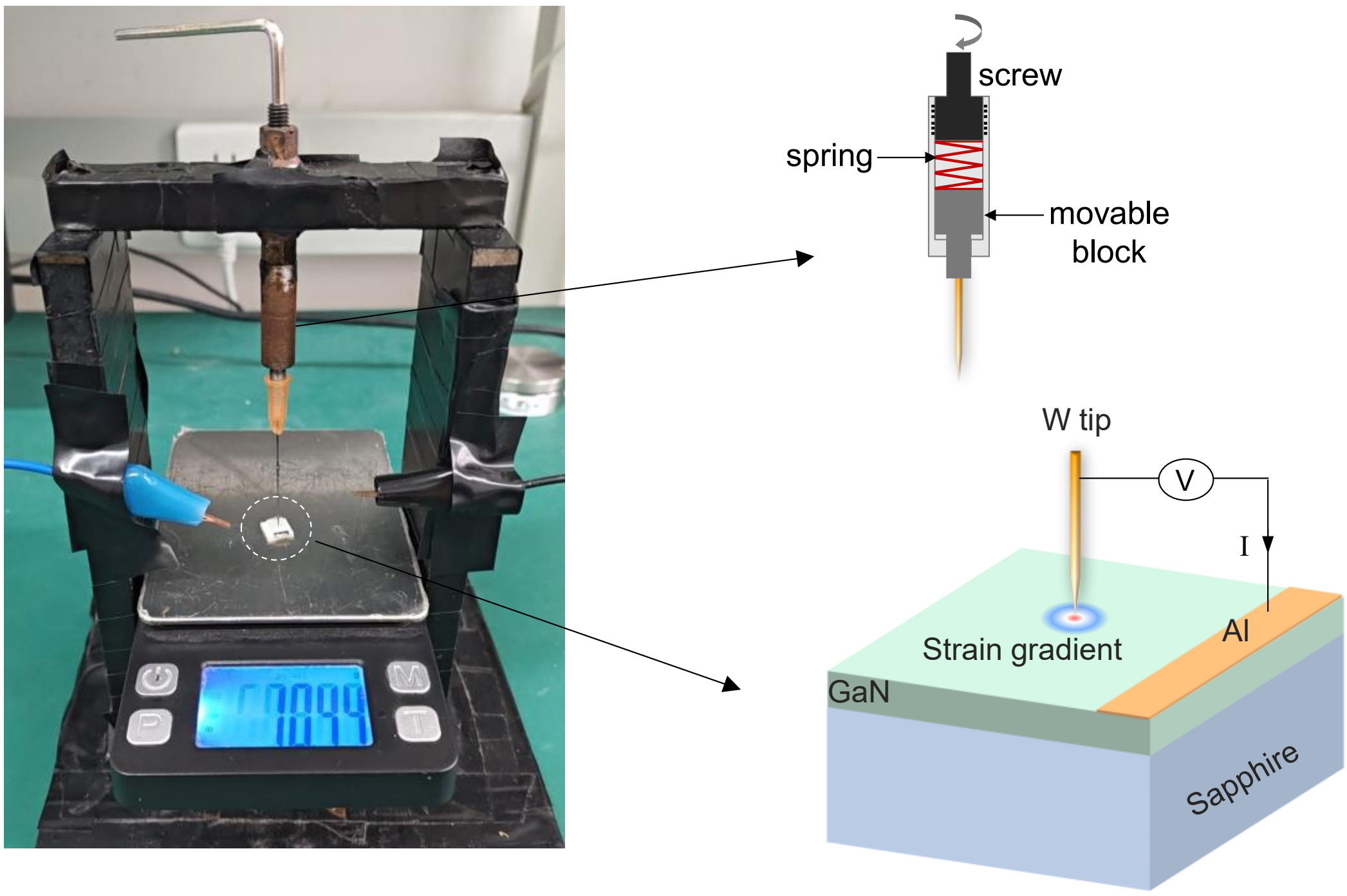

Figure S3. The home-made tip-loading stage for measurement. The tungsten conducting tip is fixed beneath the movable punch. The other end of the punch is connected to a flexible spring and a vertically adjustable screw. Rotating the screw compresses the spring, generating a controllable static normal force on the tip. A highly sensitive force sensor directly measures the load on the tip.

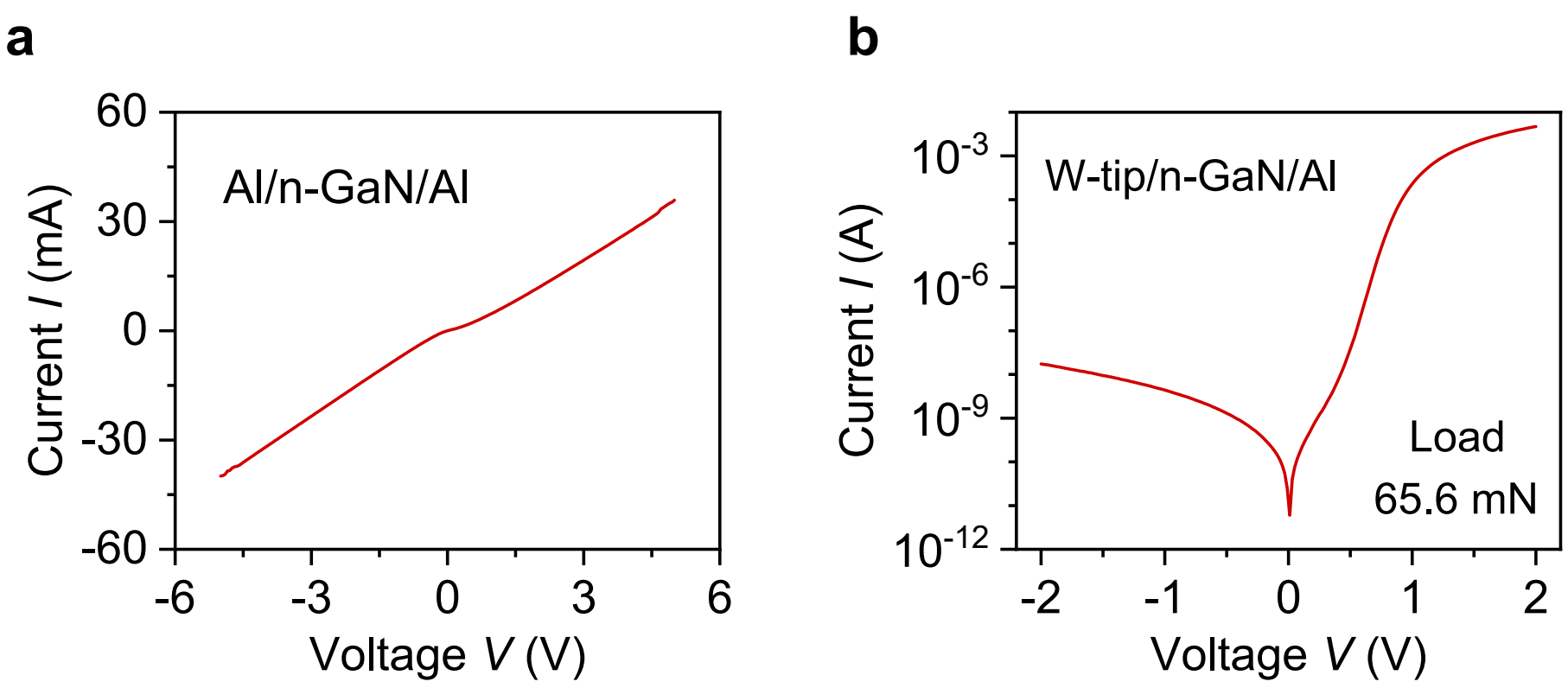

Figure S4. The contact types of n-GaN device. (a) Ohmic contact of Al/n-GaN/Al, and (b) Schottky contact of W-tip/n-GaN/Al (load a tip load of 65.6 mN).

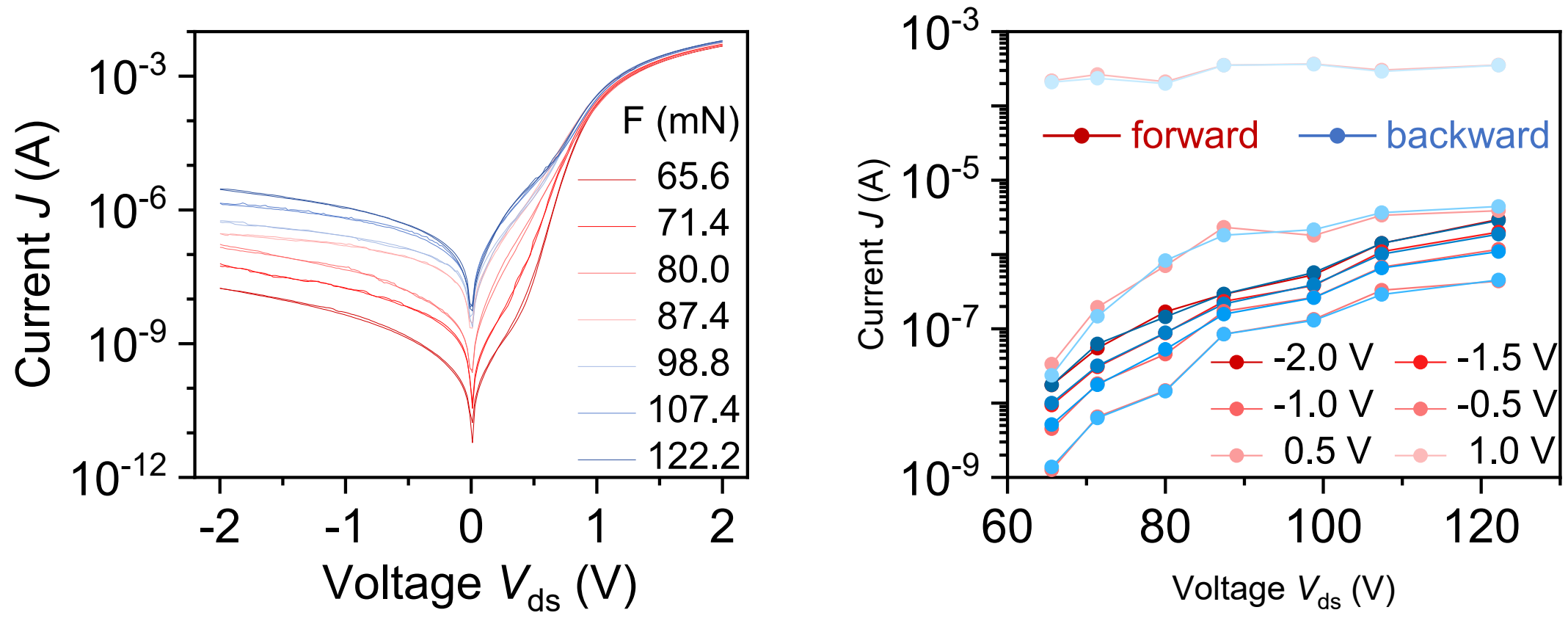

Figure S5. Reversibility of bias-dependent sensitivity switching in SGPT. The forward and backward bias-sweep I–V characteristics measured under different applied loads using a 50 μm-radius spherical tip, together with extracted I–load curves at representative bias voltages from −2 V to 1 V. The I–V curves and I–load responses show good overlap between the two sweeping directions, indicating negligible hysteresis and reversible switching between high- and low-sensitivity states.

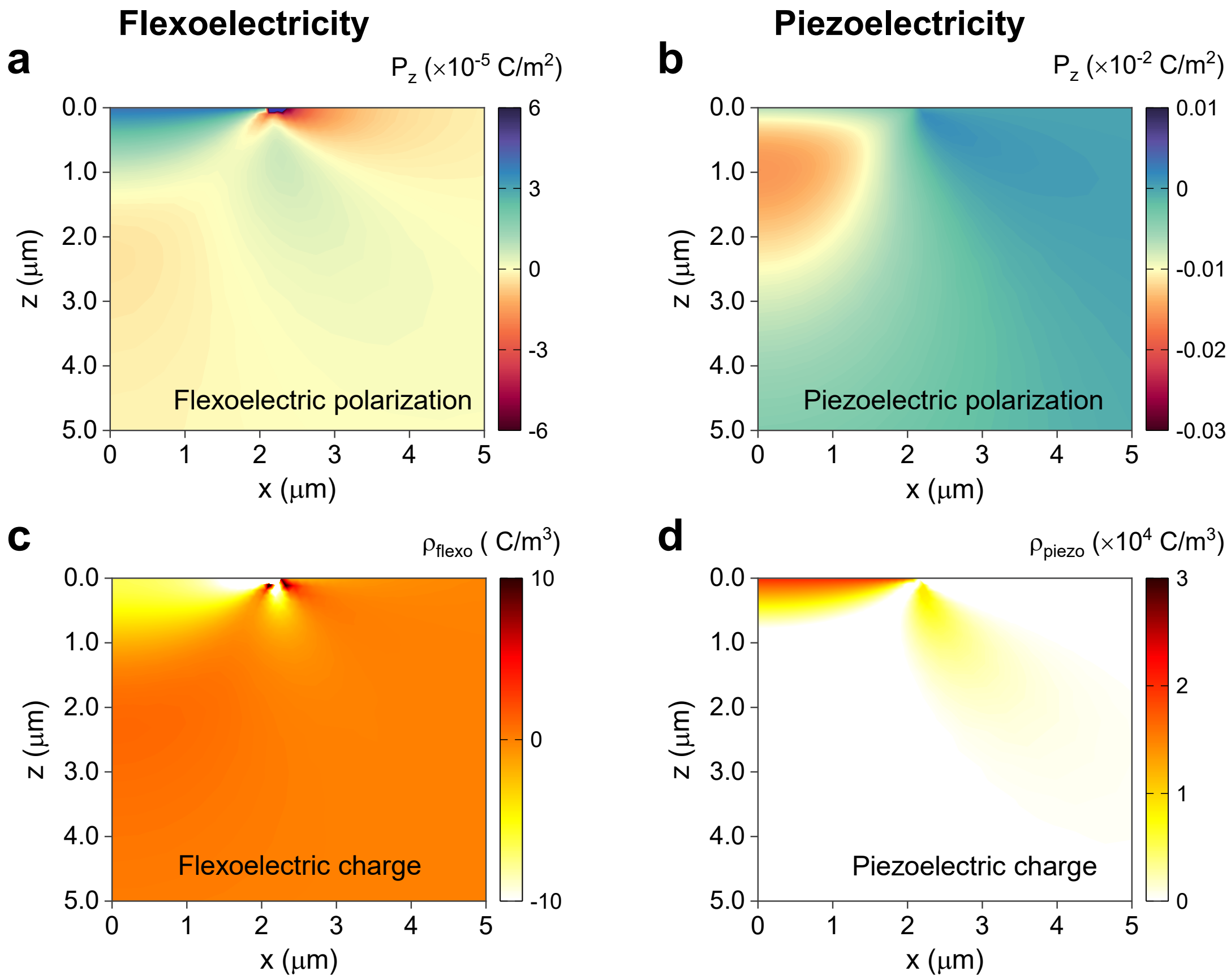

Figure S6. The comparison of flexoelectricity and piezoelectricity in GaN film under a tip load. (a) flexoelectric polarization P_z, (b) piezoelectric polarization P_z, (c) flexoelectric charge, and (d) piezoelectric charge. Here, the tip radius is R = 50 μm under a load of 100 mN. Since the absence of reported flexoelectric coefficients in GaN, we replace them with Si.

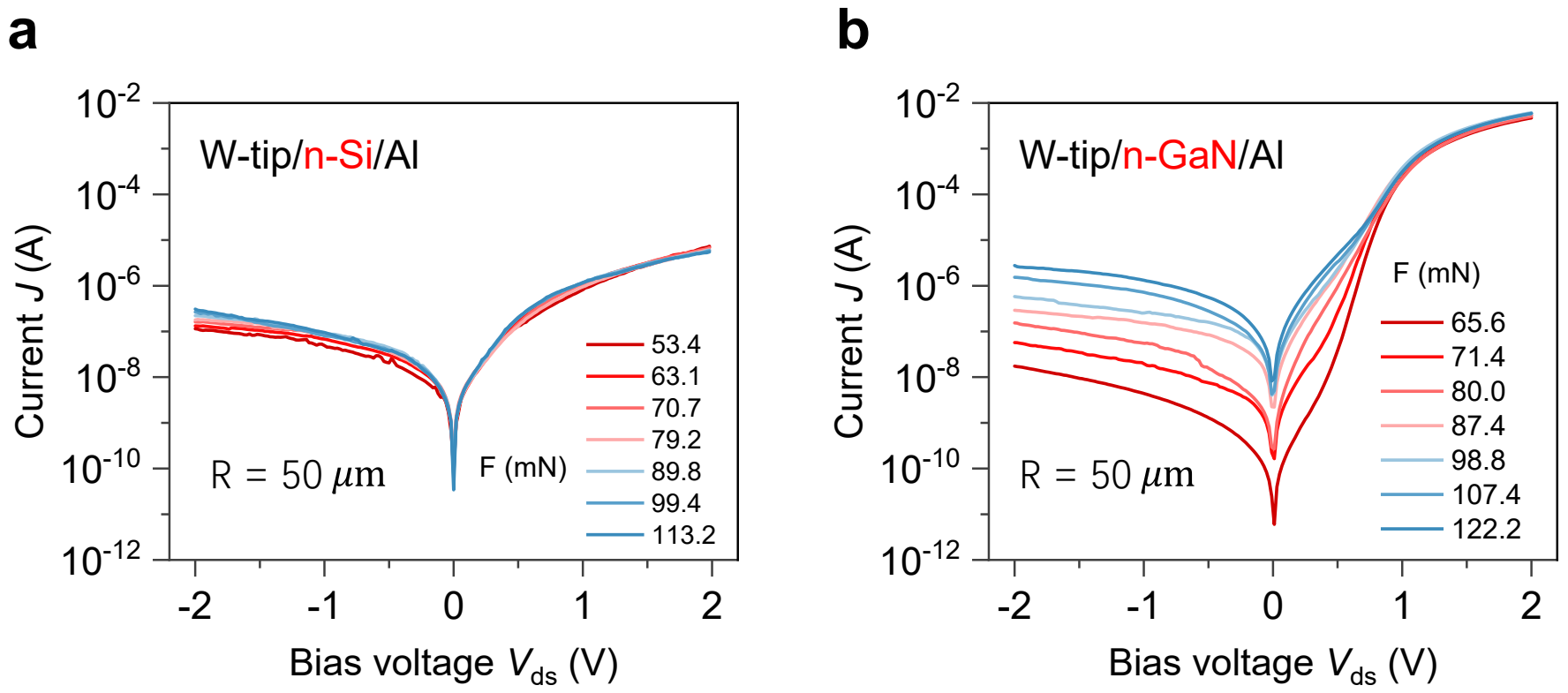

Figure S7. The comparison of strain-gradient flexoelectronic and piezotronic effect. (a) the flexoelectronic transistor based on W-tip/n-Si/Al, and (b)the piezotronic transistor of W-tip/n-GaN/Al. Here, the tip radius is R = 50 μm. Here, Si is non-piezoelectric material with strain gradient that induces flexoelectric polarization to modulate electron transport. Within near the same range of tip load, the electric response of n-Si is much lower than that of n-GaN case. This is due to the low strain gradient (~ 10^4 m^{-1}) at microscale tip and small flexoelectric coefficient for Si [6].

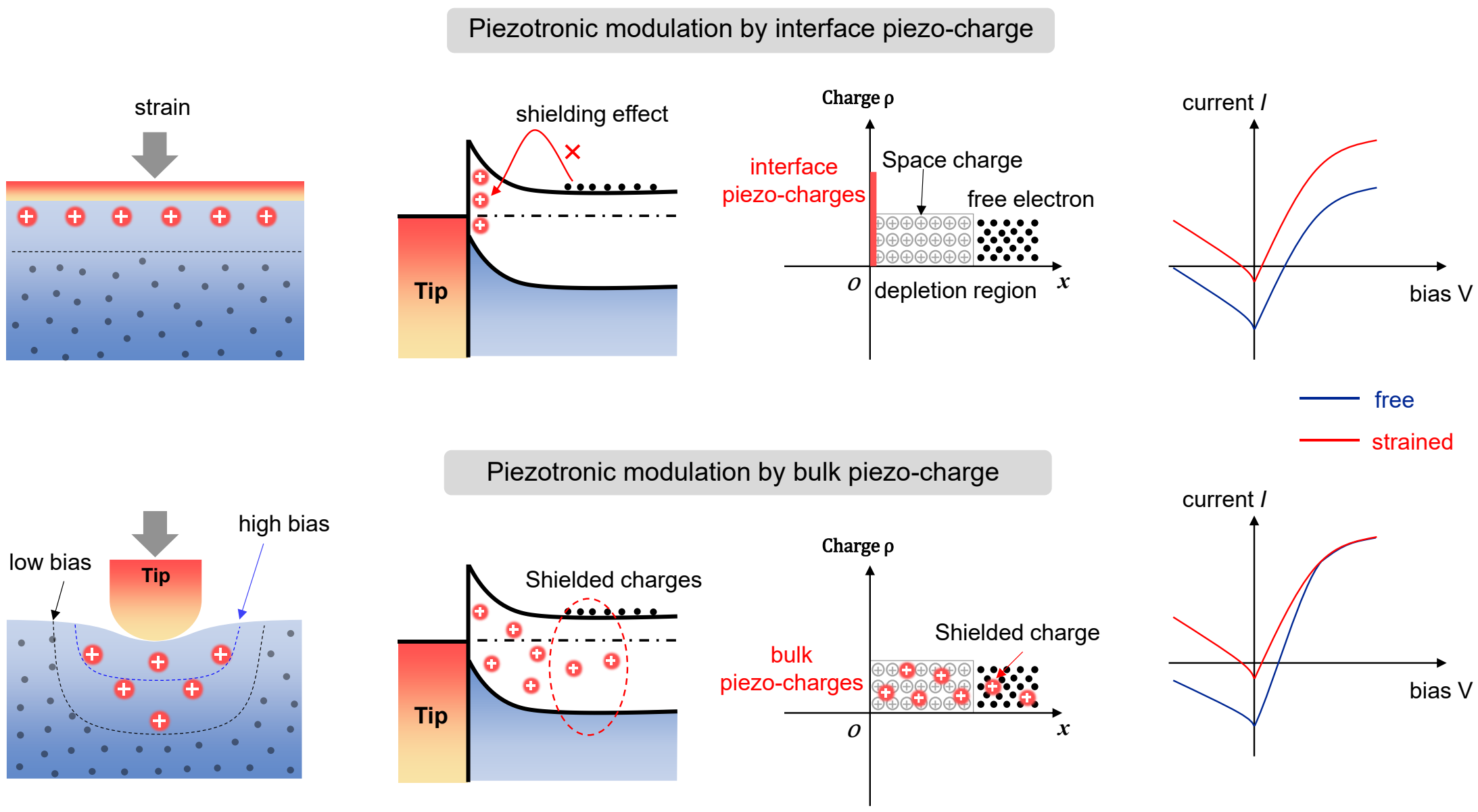

Figure S8. Schematic comparison of shielding behaviors between interface and bulk piezo-charges. In interface-dominated piezotronic modulation, piezo-charges induced by uniform strain are mainly accumulated at the junction interface and spatially separated from mobile carriers by the depletion region, resulting in weak direct charge compensation and limited bias-dependent shielding and current-voltage modulation. In the strain-gradient piezotronic transistor, bulk piezo-charges are distributed inside GaN beneath the tip contact. Bulk piezo-charges populate outside of depletion region, and bias-dependent shielding effect.

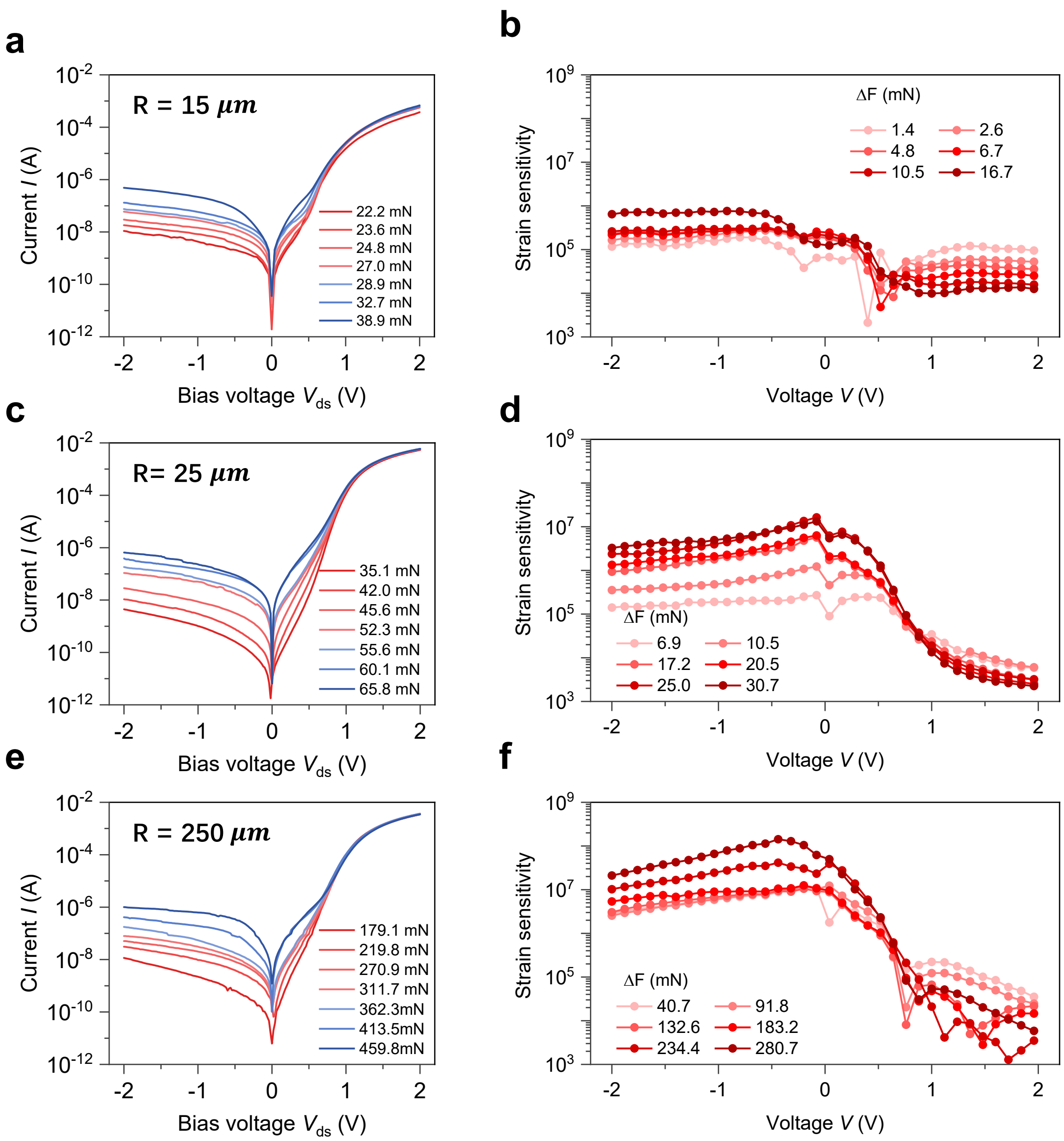

Figure S9. The size effect on the strain-gradient piezotronic transistors. The current-voltage characteristics for spherical tip with radius (a) R = 15 μm, (c) R = 25 μm, and (e) R = 250 μm. The corresponding strain sensitivity are shown for (b) R = 15 μm, (d) R = 25 μm, and (f) R = 250 μm. All devices show the piezotronic response and strain sensitivity depending on bias voltage. The peak of strain sensitivity increases with growing tip radius. The maximum values of strain sensitivity and tunability are respectively 1.43×10^8 and 24,403 for R = 250 μm at load variation of 280.7 mN.

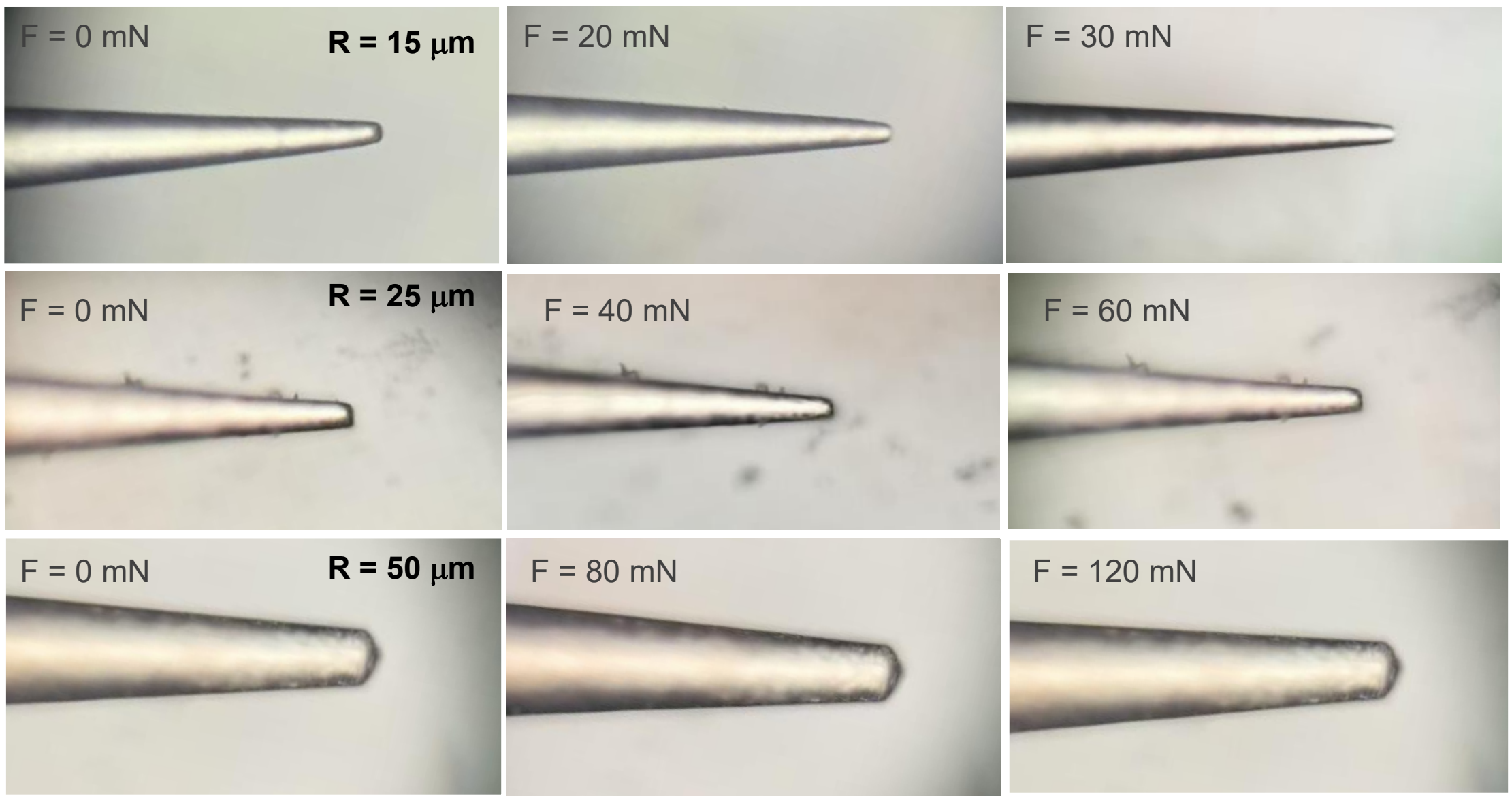

Figure S10. The optical microscope photographs of spherical tungsten tips before and after load. Here, the tips have indenter radius of R = 15, 25 and 50 μm.

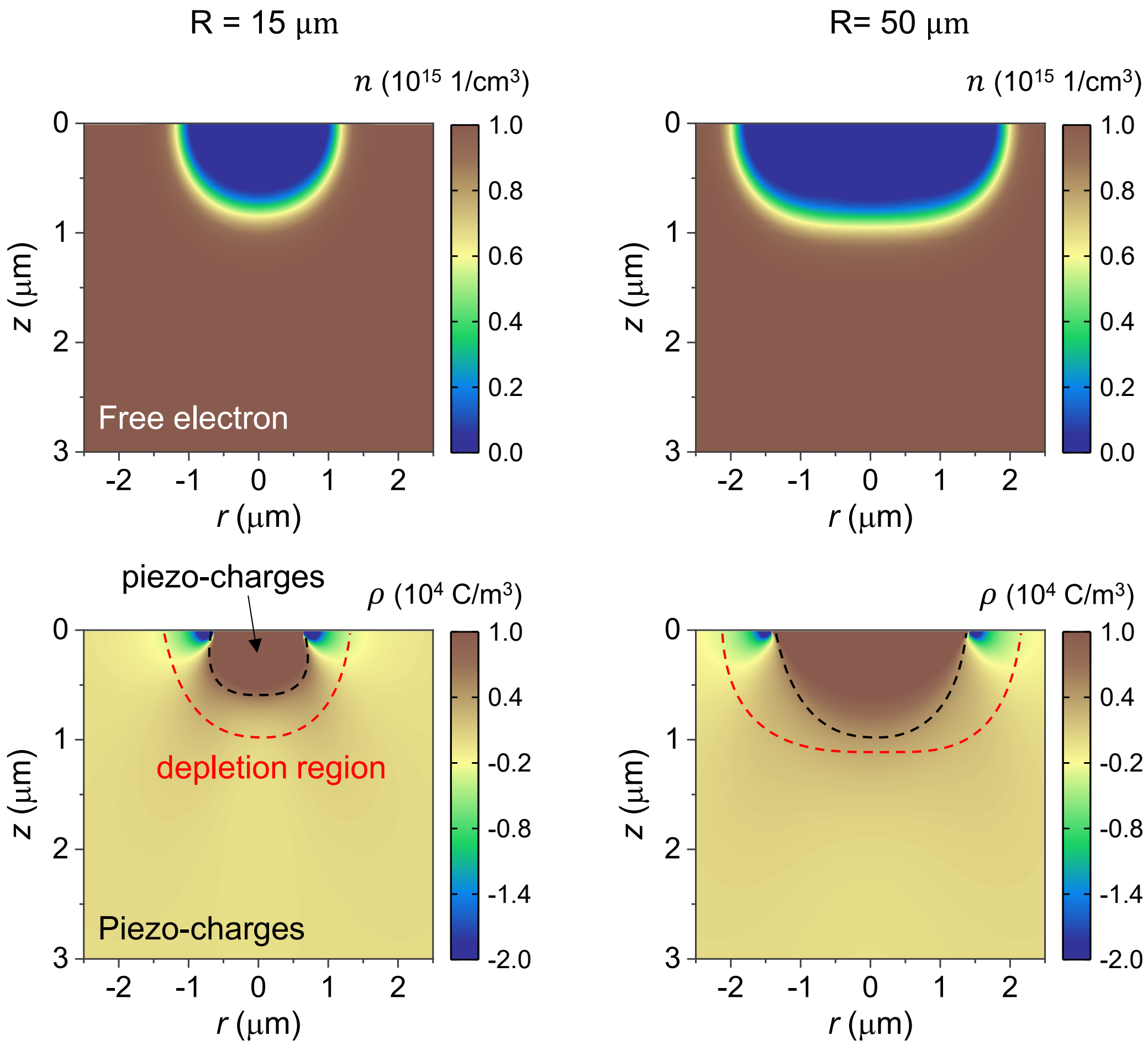

Figure S11. Quantitative comparison of the depletion region and piezo-charge distribution for different tip radii. COMSOL-simulated electron concentration maps and piezo-charge distributions in n-GaN for R = 15 μm under 10 mN and R = 50 μm under 30 mN. The depletion region shows a comparable depth for the two tip radii, whereas the larger tip produces a broader piezo-charge distribution within the electron-depleted region, leading to enhanced polar-region occupation and stronger Schottky barrier modulation.

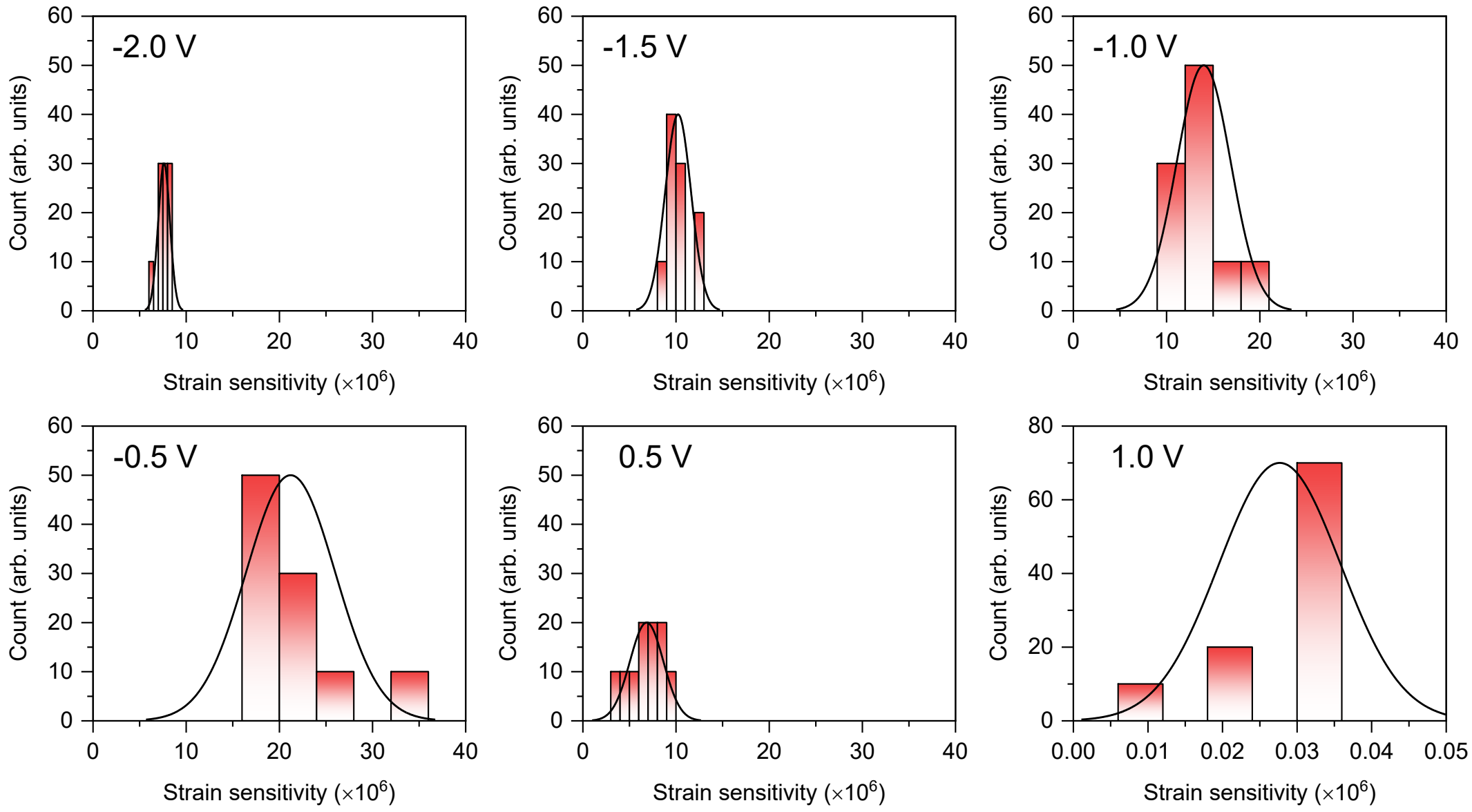

Figure S12. Device-to-device reproducibility of strain-gradient piezotronics transistor. Strain sensitivity statistics of ten strain-gradient piezotronics transistor devices measured under different bias voltages using a 50 μm-radius spherical tip, with the reference load fixed at 66 mN and the testing load at 122 mN. The devices exhibit good consistency from −2 V to 1 V, confirming the reproducibility of the bias-dependent sensitivity modulation.

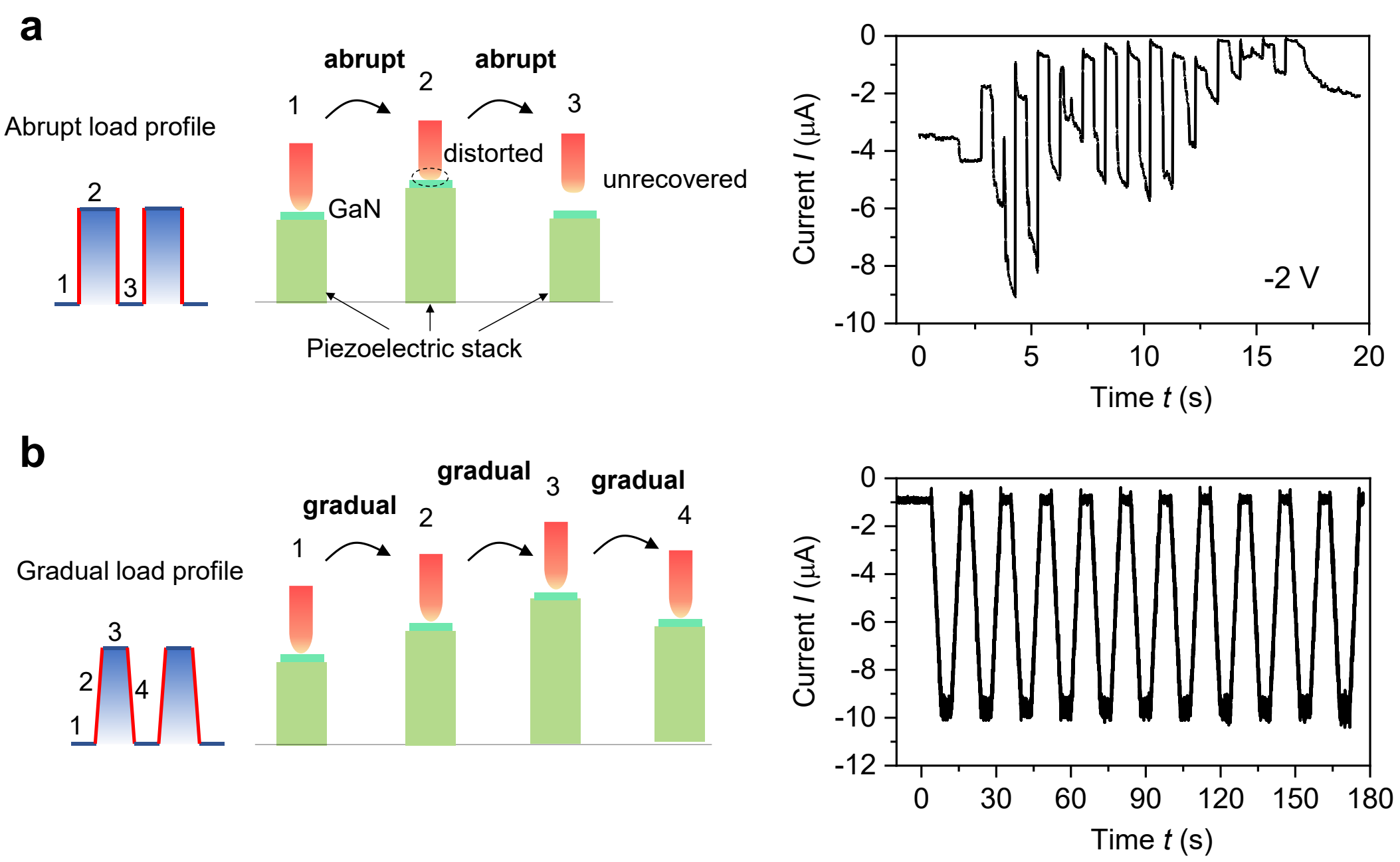

Figure S13. The impact of force waveform profile on dynamic test for strain-gradient piezotronic transistors. A square-wave load is generated by a piezoelectric stack with an abrupt step (a) and a gradual step (b). The abrupt displacement of the piezoelectric stack can cause the indenter to lag behind the actuator motion, potentially leading to unintended deformation in tip during the extension stage (1→2) and incomplete contact with the GaN surface during the subsequent retraction stage (2→3). By contrast, a gradually varying load profile effectively avoids this issue, ensuring continuous and well-controlled contact between the tip and the GaN surface. To verify this, we measured the device current at a bias of –2 V using a spherical tip of R=50 μm under a dynamic load amplitude of 40 mN. The results confirm that the slow-ramp force waveform enables highly stable operation throughout the measurement cycle.

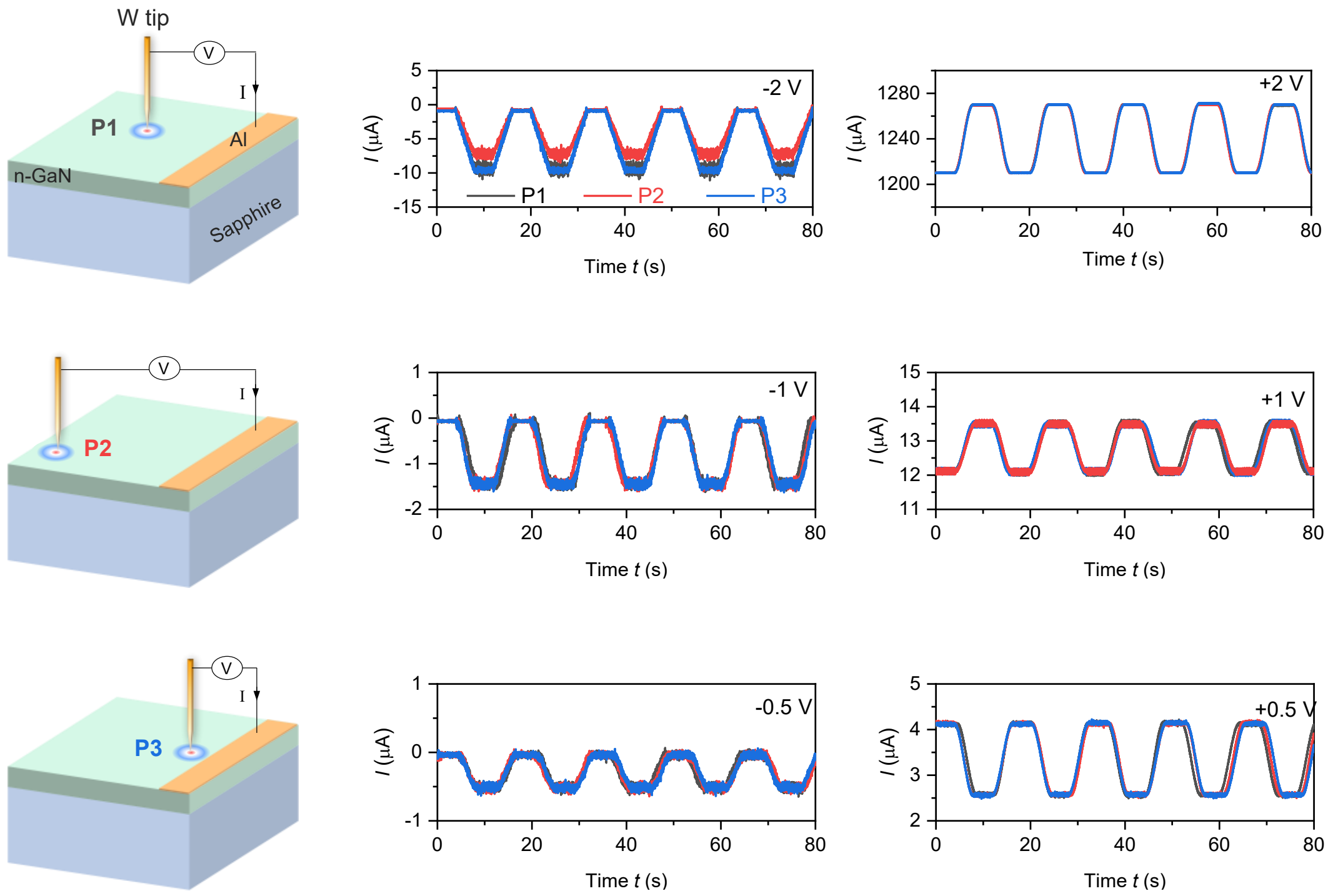

Figure S14. The repeatability of dynamic response for strain-gradient piezotronic transistors. Here, the time-dependent output current is test by putting the tip at three typical positions (P1, P2 and P3). Under forward and reverse bias, the current at three position shows highly consistence, suggesting the excellent repeatability of tip-based piezotronic transistor.

Reference

[1] F.F. Ling, Mechanical Engineering Series, Springer, 2006.

[2] L. Shu, X. Wei, T. Pang, X. Yao, C. Wang, Symmetry of flexoelectric coefficients in crystalline medium, Journal of Applied Physics. 110, (2011).

[3] P.V. Yudin, A.K. Tagantsev, Fundamentals of flexoelectricity in solids, Nanotechnology. 24, 432001 (2013).

[4] C.A. Mizzi, A.Y. Lin, L.D. Marks, Does flexoelectricity drive triboelectricity?, Phys. Rev. Lett. 123, 116103 (2019).

[5] I. Vurgaftman, J.R. Meyer, L.R. Ram-Mohan, Band parameters for III–V compound semiconductors and their alloys, Journal of applied physics. 89, 5815-5875 (2001).

[6] J. Hong, D. Vanderbilt, First-principles theory and calculation of flexoelectricity, Physical Review B—Condensed Matter and Materials Physics. 88, 174107 (2013).

[7] N. Aggarwal, S.T. Krishna, L. Goswami, M. Mishra, G. Gupta, K. Maurya, S. Singh, N. Dilawar, M. Kaur, Extenuation of stress and defects in GaN films grown on a metal–organic chemical vapor deposition-GaN/c-sapphire substrate by plasma-assisted molecular beam epitaxy, Crystal Growth & Design. 15, 2144-2150 (2015).

[8] C. Chen, Q. Yu, S. Liu, Y. Qin, Piezotronic Transistors Based on GaN Wafer for Highly Sensitive Pressure Sensing with High Linearity and High Stability, ACS nano. 18, 13607-13617 (2024).

[9] G. Hu, Y. Zhang, Y. Chao, F. Huang, M. Li, M. Liu, W. Huang, Tunable sensitivity of strain sensor by coupling piezotronic and tunneling effects, Nano Energy. 111440 (2025).

[10] S. Pan, Z. Liu, M. Wang, Y. Jiang, Y. Luo, C. Wan, D. Qi, C. Wang, X. Ge, X. Chen, Mechanocombinatorially screening sensitivity of stretchable strain sensors, Adv. Mater. 31, 1903130 (2019).

[11] Z. Wang, S. Wang, B. Lan, Y. Sun, L. Huang, Y. Ao, X. Li, L. Jin, W. Yang, W. Deng, Piezotronic Sensor for Bimodal Monitoring of Achilles Tendon Behavior, Nano-Micro Letters. 17, 1-12 (2025).

[12] J.-H. Pu, X. Zhao, X.-J. Zha, W.-D. Li, K. Ke, R.-Y. Bao, Z.-Y. Liu, M.-B. Yang, W. Yang, A strain localization directed crack control strategy for designing MXene-based customizable

sensitivity and sensing range strain sensors for full-range human motion monitoring, Nano Energy. 74, 104814 (2020).
[13] X.-C. Tan, J.-D. Xu, J.-M. Jian, G.-H. Dun, T.-R. Cui, Y. Yang, T.-L. Ren, Programmable sensitivity screening of strain sensors by local electrical and mechanical properties coupling, ACS nano. 15, 20590-20599 (2021).
[14] Y. Luo, X. Chen, X. Li, H. Tian, S. Li, L. Wang, J. He, Z. Yang, J. Shao, Heterogeneous strain distribution based programmable gated microchannel for ultrasensitive and stable strain sensing, Adv. Mater. 35, 2207141 (2023).
[15] Y. Jiang, Z. Liu, N. Matsuhisa, D. Qi, W.R. Leow, H. Yang, J. Yu, G. Chen, Y. Liu, C. Wan, Auxetic mechanical metamaterials to enhance sensitivity of stretchable strain sensors, Adv. Mater. 30, 1706589 (2018).
[16] H. Jing, J. Dan, H. Wei, T. Guo, Z. Xu, Y. Jiang, Y. Liu, Sign-Switchable Poisson's Ratio Design for Bimodal Strain-to-Electrical Signal Transducing Device, Adv. Mater. 37, 2413774 (2025).
[17] Z. Wang, C. Liang, J. Sun, Y. Yang, J. Sun, G. Tian, D. Yang, Q. Zhao, H. Liu, C. Ma, Double-Layered Microcracks Coupled Strain Sensors with High Sensitivity and Wide Working Range, Small. 2412321 (2025).
[18] F. Huang, Y. Chao, Q. Yang, M. Dan, Q. Chen, G. Hu, W. Huang, Piezotronic strain sensor with uniform and switchable sensitivity by conductivity transformation, Nano Energy. 134, 110535 (2025).
[19] G. Hu, L. Zeng, F. Huang, S. Fan, Q. Chen, W. Huang, A switchable high-sensitivity strain sensor based on piezotronic resonant tunneling junctions, Nano Research. 17, 10242-10254 (2024).
[20] Q. Yu, R. Ge, J. Wen, T. Du, J. Zhai, S. Liu, L. Wang, Y. Qin, Highly sensitive strain sensors based on piezotronic tunneling junction, Nature communications. 13, 778 (2022).
[21] T. Nguyen, T. Dinh, A.R.M. Foisal, H.-P. Phan, T.-K. Nguyen, N.-T. Nguyen, D.V. Dao, Giant piezoresistive effect by optoelectronic coupling in a heterojunction, Nature communications. 10, 4139 (2019).
[22] L. Wang, S. Liu, X. Feng, C. Zhang, L. Zhu, J. Zhai, Y. Qin, Z.L. Wang, Flexoelectronics

of centrosymmetric semiconductors, Nature nanotechnology. 15, 661-667 (2020).

[23] D. Guo, P. Guo, L. Ren, Y. Yao, W. Wang, M. Jia, Y. Wang, L. Wang, Z.L. Wang, J. Zhai, Silicon flexoelectronic transistors, Science advances. 9, eadd3310 (2023).

[24] P. Keil, M. Trapp, N. Novak, T. Frömling, H.J. Kleebe, J. Rödel, Piezotronic tuning of potential barriers in ZnO bicrystals, Adv. Mater. 30, 1705573 (2018).

[25] S. Liu, L. Wang, X. Feng, Z. Wang, Q. Xu, S. Bai, Y. Qin, Z.L. Wang, Ultrasensitive 2D ZnO piezotronic transistor array for high resolution tactile imaging, Adv. Mater. 29, 1606346 (2017).

[26] S. Liu, L. Wang, Z. Wang, Y. Cai, X. Feng, Y. Qin, Z.L. Wang, Double-channel piezotronic transistors for highly sensitive pressure sensing, ACS nano. 12, 1732-1738 (2018).

[27] X. Han, W. Du, R. Yu, C. Pan, Z.L. Wang, Piezo-Phototronic Enhanced UV Sensing Based on a Nanowire Photodetector Array, Advanced Materials (Deerfield Beach, Fla.). 27, 7963-7969 (2015).

[28] Y.S. Zhou, K. Wang, W. Han, S.C. Rai, Y. Zhang, Y. Ding, C. Pan, F. Zhang, W. Zhou, Z.L. Wang, Vertically aligned CdSe nanowire arrays for energy harvesting and piezotronic devices, ACS nano. 6, 6478-6482 (2012).

[29] S. Das, B. Wang, T.R. Paudel, S.M. Park, E.Y. Tsymbal, L.-Q. Chen, D. Lee, T.W. Noh, Enhanced flexoelectricity at reduced dimensions revealed by mechanically tunable quantum tunnelling, Nature communications. 10, 537 (2019).

[30] X. Jiang, X. Wang, X. Wang, X. Zhang, R. Niu, J. Deng, S. Xu, Y. Lun, Y. Liu, T. Xia, Manipulation of current rectification in van der Waals ferroionic CuInP2S6, Nature communications. 13, 574 (2022).
